# Changing dynamics of *Aedes aegypti* invasion and vector-borne disease risk for rural communities in the Peruvian Amazon

**DOI:** 10.1371/journal.pntd.0012506

**Published:** 2025-08-28

**Authors:** Kara Fikrig, Arnold O. Noriega, Rosa A. Rodriguez, John Bardales, José Rivas, Becker Reyna, Guido Izquierdo, Gissella M. Vasquez, Ryan T. Larson, Amy C. Morrison, Laura C. Harrington

**Affiliations:** 1 Cornell University, Ithaca, New York, United States of America; 2 Asociación Benéfica Prisma, Lima, Perú; 3 Gerencia Regional de Salud Loreto, Iquitos, Perú; 4 U.S. Naval Medical Research Unit SOUTH, Lima, Perú; 5 University of California Davis, Davis, California, United States of America; Egerton University, KENYA

## Abstract

*Aedes aegypti,* the primary vector of dengue virus, is predominantly considered an urban mosquito, especially in the Americas, where its reemergence began in cities after the end of continent-wide eradication campaigns. The results of our study diverge from this narrative, demonstrating the recent and widespread rural invasion of *Ae. aegypti* along major shipping routes in the northern Peruvian Amazon between the major cities of Iquitos, Pucallpa, and Yurimaguas. Using prokopack aspirators to conduct indoor mosquito collections, we identified *Ae. aegypti* populations in 29 of 30 sites surveyed across a rural to urban gradient and quantified *Ae. aegypti* adult metrics. In multiple instances, adult *Ae. aegypti* indices in rural villages were equal to or greater than indices in dengue-endemic cities, suggesting the entomological risk level in some rural areas is sufficient to support dengue transmission. Fourteen rural sites were sampled in transects from the community river port into town. In seven of these sites, houses closer to the port were significantly more likely to be infested with *Ae. aegypti* adults than houses further from the ports, and four additional sites showed a similar trend. This pattern suggests that *Ae. aegypti* is still actively invading many rural sites by adult *Ae. aegypti* disembarking from boats at the port, finding nearby oviposition sites, and advancing stepwise towards the interior, with sections of towns still *Ae. aegypti-*free. Only one site showed a strong signal of invasion via the egg or larval stage, with a focus of *Ae. aegypti* far removed from the port. The widespread infestation of *Ae. aegypti* in rural areas is a major public health threat given the far distance of communities to hospital care. It is important to implement control measures now before the mosquito gains a stronger foothold in zones of active invasion.

## Author summary

The dengue virus and its mosquito vector, *Aedes aegypti,* have historically been considered an urban problem in South America. Since its reemergence following the continent-wide eradication campaign in the mid-1900s, *Ae. aegypti* has predominantly infested urban areas, leaving rural areas relatively unaffected by the arboviruses it transmits. This study provides strong evidence that this paradigm is shifting in the Peruvian Amazon. We identified *Ae. aegypti* infestations in 29/30 sites spanning a spectrum of urbanization, from villages to cities. Infestation metrics (proportion of infested houses and average number of adult *Ae. aegypti* per house) were higher in some villages than certain cities. We observed a repeated pattern of *Ae. aegypti* populations clustered near village ports, suggesting that the invasions often occur when adult mosquitoes fly off boats at port and subsequent generations spread house-by-house inland from the entry point. This pattern also indicates that some rural communities are experiencing ongoing invasions, and that rural infestations of *Ae. aegypti* will likely continue to expand as newly invaded populations become fully established. This shift in *Ae. aegypti* ecology in the Peruvian Amazon will have severe public health implications, increasing the risk of dengue and other arboviruses for remote villages with limited access to healthcare.

## Introduction

*Aedes aegypti* mosquitoes transmit numerous viruses impacting human health, including dengue virus, across the global tropics and subtropics. Dengue virus causes about 400 million infections per year, resulting in over 40,000 deaths [1,2]. The disease can manifest a significant range of morbidity, including the need for advanced hospital care in the ICU, leading to a cumulative cost of about US$8.9 billion [1,3].

Dengue was first reported in Peru in 1990, following closely on the heels of the reemergence of *Ae. aegypti* in the country. The mosquito was declared eradicated in Peru in 1958, as a result of the continent-wide Yellow Fever eradication campaign [4,5]. In 1984, *Ae. aegypti* was detected once again in Iquitos, an Amazonian city only accessible by boat and plane [5]. In the span of three years, *Ae. aegypti* quickly reinvaded the city, increasing infestation levels from 1% to 26% by 1988 [5]. The first reported dengue outbreak in Iquitos occurred soon thereafter [5]. Similar patterns of *Ae. aegypti* eradication, reinfestation, increased dispersion, and dengue transmission repeated across much of the Americas during a similar time frame [6].

Because *Ae. aegypti* reinfestation and dengue transmission in the Americas began in densely populated urban areas, the vector and virus have been considered an urban problem [7]. Recently, the geographic profile of *Ae. aegypti* distribution and dengue transmission in the Americas has begun to change, expanding to more remote, rural communities [8]. There have been increasing reports of rural *Ae. aegypti* populations in numerous countries across the continent [9–13], which will likely expand further with increasing levels of transport connectivity, increasing use of plastics and other waste that can serve as larval habitat in rural areas, and persistent lack of access to running water, necessitating water collection [8]. This trend has received limited attention despite mirrored increases in rural dengue transmission, with evidence of high seroprevalence and the circulation of multiple serotypes in rural communities, particularly in Colombia and Ecuador [14–17]. Importantly, records of this rural expansion of the mosquito and virus is likely a substantial underestimate of their true rural distribution due to limited vector and disease surveillance in rural areas.

The northern Peruvian Amazon is an interesting setting to explore the expansion of *Ae. aegypti* given the heterogeneity of urbanization and fluvial connectivity throughout the region. Iquitos, the city where dengue was first identified in Peru, is connected by river to the port cities of Pucallpa and Yurimaguas, which also have endemic dengue transmission and established *Ae. aegypti* populations [18–20]. The rivers connecting these cities serve as a fluvial highway to transport goods and people between the cities, as well as the hundreds of rural communities scattered along the riverbanks.

The boats that travel these rivers play a critical role in *Ae. aegypti* mosquito dissemination to remote communities [21,22]. A comprehensive survey of terrestrial and fluvial vehicles showed that all forms of fluvial transit could be infested with *Ae. aegypti,* with the highest infestation rate in large cargo boats (71.9%) [23]. Cargo boats were even found to sustain active oviposition by *Ae. aegypti,* allowing the mosquito to complete its full life cycle aboard the ship [24].

The first detection of *Ae. aegypti* in rural communities outside of Iquitos was in 2008, through an epidemiological study on arboviruses conducted by the U.S. Naval Medical Research Unit SOUTH (then known as the Navy Medical Research Center Detachment, NMRCD) and independent routine surveillance activities conducted by the Peruvian Ministry of Health [21]. In 2011–2012, a more comprehensive *Ae. aegypti* survey was conducted across 34 sites along the Amazon River and the 95 km stretch of road between Iquitos and Nauta (a small nearby city) [21]. These collections showed heterogeneous mosquito distributions in riverine communities and spatially explicit patterns along the roads. Half of surveyed riverine communities were infested. Communities with a larger human population size and closer distance to Iquitos were more likely to be infested with *Ae. aegypti.* Collections along the road showed presence of *Ae. aegypti* in all communities up to a discrete point, after which all further communities were negative. At the time, the furthest point of *Ae. aegypti* expansion from Iquitos was 37.1 km by river and 19.3 km by road [21].

In the intervening time, there has been no further systematic characterization of rural *Ae. aegypti* populations in the Peruvian Amazon, and no characterization of the mosquito beyond 95 km from Iquitos. The lack of information regarding vector movement and presence in remote areas is particularly concerning because communities in these areas are located far from medical facilities and care required to treat severe dengue. A combination of remote location and insufficient resources have created highly inequitable access to health care for rural communities in the Peruvian Amazon [25,26].

Basic ecological information about the distribution of *Ae. aegypti* is vital to understand rural dengue risk in this region. To address this need, we conducted mosquito collections in thirty sites spread across a wide expanse of the northern Peruvian Amazon along over 1,000 km of river, across a rural – urban gradient, to determine the distribution of *Ae. aegypti* and measure the relative entomological risk for transmission of *Aedes-*borne viruses in the region. We expected to observe a trend in infestation levels associated with the degree of urbanization, anticipating that more populous communities would be more likely to be infested and exhibit higher entomological indices. Indeed, we did detect a trend of this nature, but it was weaker than expected because of the near ubiquity of *Ae. aegypti* across the transect. We detected *Ae. aegypti* infestations in almost all sites surveyed and measured high entomological indices in numerous small rural communities, suggesting that the expansion process is more advanced than previously imagined.

## Methods

### Ethics statement

All necessary permits and approvals were obtained, including the SERFOR collection permit (RD N°000066-2024-MIDAGRI-SERFOR-DGGSPFFS-DGSPFS), Gerencia Regional de Salud approval (N° 256-2022-GRL-DRSL/30.09-INVESTIGACIÓN), and household collections were deemed exempt by the institutional review boards (IRB) of the U.S. Naval Medical Research Unit SOUTH (NAMRU6.P0003) and Cornell University (IRB0145012). Formal verbal consent was obtained from the communities’ leadership as well as from each engaged household (see S1 Text).

In the following sections, we describe the rationale we used to select our sites, followed by the characteristics of the selected sites, mosquito collection methods, geographic data details, and statistical analysis methods. We then finish with statements on data availability, permits, and the availability of a Spanish translation of this paper.

### Site selection

**Fig 1:**
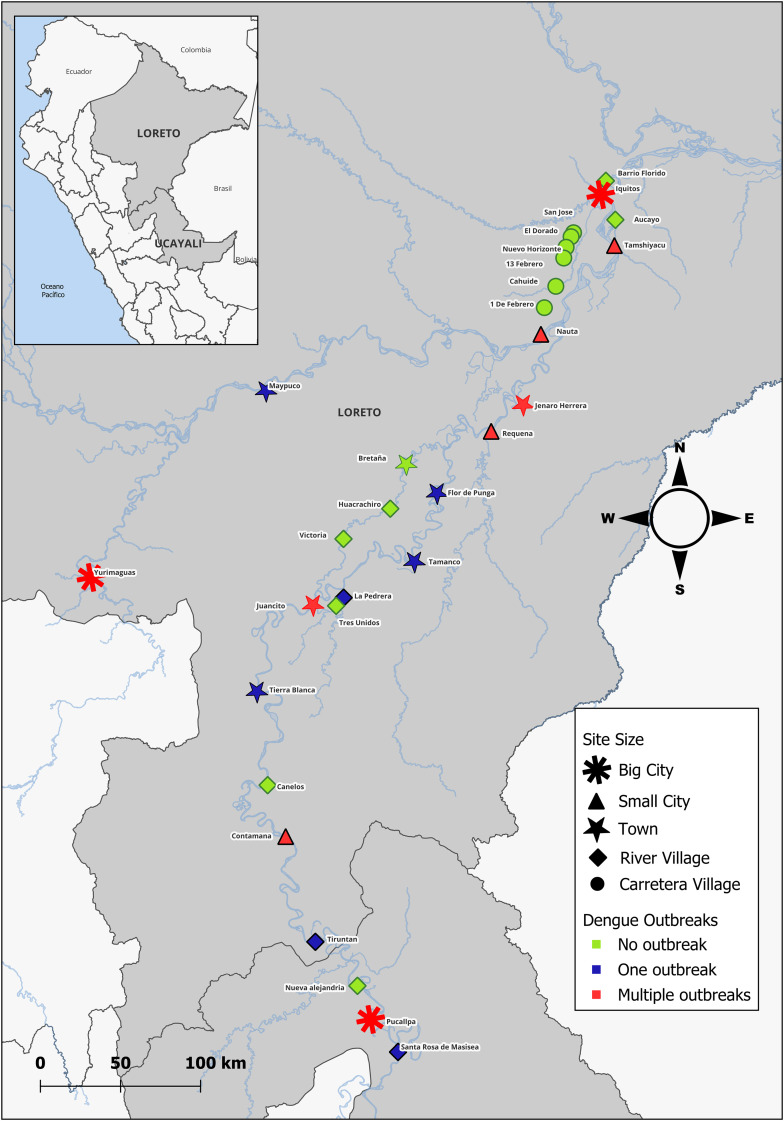
Map of sampling sites (30) across the regions of Loreto and Ucayali, demonstrating site size in symbols (black in legend) and history of dengue outbreaks in color (square in legend). Map created in qGIS with shape files from the Peruvian Government [27] and the United Nations Office for the Coordination of Humanitarian Affairs [28].

In this study, we sought to characterize features of the invasion of *Ae. aegypti* into remote communities across a rural – urban gradient in the Loreto and Ucayali states in the Northern Peruvian Amazon. This region of the Amazon has several big and small cities and many towns and villages scattered throughout the immense expanse of rainforest. The communities are primarily connected by the complex matrix of rivers that cut through the forest. Mosquito collections were concentrated along the river system connecting Iquitos to Pucallpa, one of the two port cities that supply Iquitos with goods from outside the Amazon (Fig 1), maximizing the likelihood of cargo ship-facilitated long-distance dispersal of *Ae. aegypti*.

We aimed to select sites balanced across population size and history of confirmed dengue outbreaks (none, one outbreak, or multiple outbreaks) and spread geographically along the river transect. We used Loreto health department records about government led insecticide spray campaigns in communities during the previous five years, where a record was considered synonymous with a dengue outbreak or malaria transmission. Malaria is not endemic in the selected communities, so insecticide treatment was in response to dengue outbreaks. Where there was no record of insecticide-use, there was no *a priori* record of *Ae. aegypti* presence. Final site selection was based on synthesis of census population reports, geographic location, and health department insecticide use data; however, given the vast number of rural communities, the final site list was driven largely by logistic considerations– the same selection criteria could have resulted in a different set of sites along the same transect.

Collections were conducted in two phases. Phase 1 involved more comprehensive collections, with an average of 87.24 households per site (range 45–162), compared to 25 households per site (range 13–40) in Phase 2. Eighteen sites were selected between Iquitos and Pucallpa and one site on the far side of Pucallpa, roughly evenly spaced across the transect, as well as the two cities. Phase 2 was initiated after Phase 1 to capitalize on a surplus of field time, allowing brief visits to 9 additional sites not initially planned. Phase 2 collections were shorter, involving fewer households per site due to logistical constraints, but all other methods remained consistent. Collections in Phase 2 included 6 sites along the short stretch of road that connects Iquitos to the small city of Nauta, all previously negative for *Ae. aegypti* in the 2011–12 collections by Guagliardo et al. [21], as well as one site on the Amazon River north of Iquitos and one community on the Marañon river route to the other port city, Yurimaguas, as well as the city itself. In total, samples were collected in 30 sites, with 21 sites in Phase 1 and 9 sites in Phase 2.

### Site characteristics

The sites are geographically dispersed along two main fluvial transit routes: Iquitos – Pucallpa and Iquitos – Yurimaguas. The Iquitos – Pucallpa route spans a Euclidian distance of 536 km, which translates to 1,068 km and over 36 hours of travel on the fast boats that transit the winding Ucayali, Puinahua and Amazon rivers. The Iquitos – Yurimaguas route spans a Euclidian distance of 399 km, which translates to 661 km and over 20 hours of travel on the fast boats along the Huallaga and Marañon rivers. We also sampled two communities beyond the cities along these rivers. The population range of the sites is 120–484,000 people. Three sites are considered big cities (>23,000 people), four are small cities (5,000 – 23,000 people), seven are towns (1,000–4,999 people), and sixteen are villages (<1,000 people; ten along the rivers (= river village) and six along the Iquitos-Nauta highway (=road village)).

Among the twenty-one sites surveyed in Phase 1 along the Iquitos-Pucallpa river route, seven communities never reported a dengue outbreak, six communities reported one dengue outbreak, and eight reported multiple outbreaks prior to the time of our collections (including Iquitos and Pucallpa, considered endemic for dengue). Five sites with one outbreak were initially selected as sites without a history of dengue (and no *a priori* knowledge of *Ae. aegypti* presence), but four experienced a dengue outbreak during the months between site selection and site visit and one experienced an outbreak just over five years prior, before the five-year time frame of health department data shared with our team. Among the nine sites surveyed in Phase 2, seven communities had no record of locally transmitted dengue or *Ae. aegypti* (all the road villages and the river village beyond Iquitos), one had history of one outbreak, and one had multiple dengue outbreaks (Yurimaguas, also considered endemic). See S1 Table to see the characteristics of each site.

While site urbanization level was classified by population size, other site characteristics are closely associated with these designations. The villages and towns had limited electrification, with electricity generated for about 3–4 hours per day (except for one town with full-day electricity), while the cities had full-day electricity. The houses in villages and towns tended to be made of wood boards, loosely fitted, with metal or palm leaf thatched roofs. In big cities, houses tended to be made of concrete with metal roofs, and small cities had a mixture of the two housing types. Most villages and towns did not have any form of official garbage management, except a few district capitals that had limited garbage collection and a dumping ground in the forest. Cities had more robust garbage management. While we consider villages and towns in this context to be rural, houses were notably aggregated, with agricultural lands dispersed along the river and into the forest. The yards were typically slightly larger than those in cities, but population density is not as dramatically different as rural/urban dichotomies in other regions.

### Mosquito collection

Before initiating mosquito collections, our team conducted extensive community engagement activities described in detail in S1 Text. A team of five collectors (four per site) conducted mosquito collections with Prokopack aspirators [29] operated inside structures between the hours of 07:00 and 18:00. Collections were conducted in residences, inside stores, offices, and structures of mixed used (store or office space alongside a living space). Structures were searched systematically in all spaces where collectors were permitted to enter using headlamps and the physical disturbance of all surfaces, including under furniture with the aspirators to collect both flying and resting mosquitoes. The time varied with the size and internal structure of the home; although the exact time was not recorded, it generally ranged from 5-30 minutes. Mosquitoes were maintained alive in mesh-covered 0.7L collection cups, labeled with the unique structure code. Mosquitoes were killed at the end of the day by placing cups inside a sealed bag with acetone for approximately 15 min. Specimens were sorted and all *Ae. aegypti* were separated and confirmed based on key taxonomic features with a field microscope [30]. Resource and time limitations precluded the identification of all other mosquito species. Notably, *Ae. albopictus* is not present in this part of the Peruvian Amazon and was not detected at any of the sites.

Limited larval surveys were also conducted, the methods and results of which are reported in S2 Text.

In Phase 1 collections (along the Iquitos-Pucallpa transect), the sampling approach was designed to include areas near and far from the ports and was adapted based on community size and boat schedules. In villages, a high percentage of houses were visited (37 – 93% of houses based on 2017 census household counts [31,32]) and the majority approached granted entry to our team. In towns, we collected in areas near and far from community ports; whenever possible, we collected in a transect from the town port to residences farthest away from the river. In towns, we sampled 12 – 31% of houses. In cities, we also collected close to and far from the port(s). Notably, city collections were the least representative of the entire city. The proportion of houses surveyed was low (< 0.2% of houses) and the sampling area was influenced by health department recommendations regarding where there was high *Ae. aegypti* prevalence.

In Phase 2 (communities along the Iquitos-Nauta road, the Yurimaguas river route, and downriver from Iquitos), collections were limited to a smaller number of houses (13 – 40 houses; coverage ranging between 13 – 70% for the villages, 8 – 13% for the towns, and 0.1% for the city, Yurimaguas).

We sampled from January through June 2023, with most collections corresponding to the period of higher rainfall, river levels, and dengue transmission. Each community was only visited once during the collection period.

Depending on structure size and other logistical considerations, collections were sometimes conducted by one collector, sometimes by two collectors, and rarely by three or four collectors. Five different collectors were involved throughout the study, but only three or four collectors were involved in the collections for any given site.

### Geographic data collection

At the time of sampling, a GPS point was taken at the front door of the property using the UTM Geo Map application (Y2 Tech, Indonesia). The GPS point was taken when the application-reported accuracy fell within 3m. The GPS coordinates were uploaded to qGIS (version 3.30) and overlaid with 2023 ESRI satellite images (2023 ESRI). The Euclidian distance was measured between each sampling point and the community port (identified via images taken during rainy season). In some communities, there are two separate ports for rainy and dry season, or for barges and passenger boats. In these cases, we selected the distance to the closest of the two ports for each sampling point for subsequent analyses.

### Data analysis

All analyses were performed in R version 4.3.0 [33]. A summary of all statistical models conducted can be found in S2 Table.

#### *Aedes aegypti* infestation levels.

For each community, the following metrics were calculated: adult house index (percentage of houses infested with adult *Ae. aegypti;* **AHI**) and mean adult number (average number of *Ae. aegypti* adults per house). Maps displaying infestation levels across the region were created using qGIS.

#### Impact of urbanization level on *Ae. aegypti* infestation levels.

Generalized linear mixed models (GLMM) were utilized to determine the impact of urbanization level (i.e., site size) on infestation metrics (presence of adult *Ae. aegypti* and number of adult *Ae. aegypti* per house). Post hoc pairwise comparisons were conducted with the ‘emmeans’ and ‘pairs’ functions with Bonferroni adjustment using the ‘emmeans’ package [34] to compare the estimated marginal means of the infestation metrics for each urbanization level, adjusting for other variables in the model. Urbanization was considered an ordinal categorical variable with five levels of increasing population size: road village, river village, town, small city, and big city. Notably, the road villages are not uniformly smaller than river villages but had a lower population on average and were grouped separately due to the distinct ecology of their locations.

Presence of *Ae. aegypti*. A GLMM was conducted to determine the impact of urbanization level on the probability of whether a house was negative (0) or positive (1) for at least one *Ae. aegypti* adult using the lme4 package [35] with a binomial distribution. The fixed effect was the urbanization level, and the random effects included the site, and the collector team (each unique collection team, whether an individual or combination of individuals, was considered a separate level of one collector variable).

Number of *Ae. aegypti.* Another GLMM was performed to determine the relationship between the urbanization level and number of adult *Ae. aegypti* per house. Due to overdispersion of the data, a glmmTMB model with a negative binomial distribution was used [36], with the same fixed and random effects as above.

#### [34] Impact of distance to port on infestation levels.

Generalized linear mixed models were performed on a subset of communities to better understand the impact of distance to the community port on *Ae. aegypti* infestation. Analyses were limited to riverine communities with a minimum of 60 data points, where at least 10% of houses were sampled (based on number of households in the 2017 census [31,32]), and where we conducted transects leading away from the port. This subset included 14 communities. To assess how the effect of distance from port varies by community, we used the ‘emtrends’ function [34] to estimate this effect for each community.

Presence of *Ae. aegypti*. First, we performed a GLMM with a binomial distribution to evaluate the impact of distance from the port on whether a house was positive for at least one *Ae. aegypti* adult. Fixed effects included the site, distance (km) of the house to the port, and the interaction of the two. The collector team was included as a random effect. The BOBYQA optimizer with 200,000 iterations was utilized.

Number of *Ae. aegypti.* We performed a GLMM to determine the impact of distance to the port on the number of adult mosquitoes per house, using glmmTMB with a negative binomial distribution with quadratic parameterization (nbinom2 family) to account for overdispersion of the data [36]. The same fixed and random effects were used as in the presence model. The code and data for these analyses have been made freely available [37].

### Spanish translation

This paper has been translated into Spanish to improve accessibility in the region where the research was conducted (see S3 Text).

## Results

### *Ae. aegypti* infestation levels

**Fig 2:**
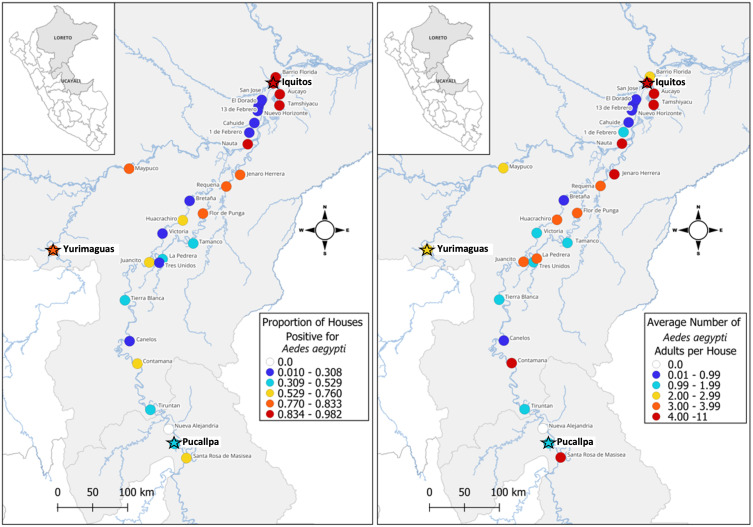
Maps of adult *Ae. aegypti* infestation levels in our sampling sites across the regions of Loreto and Ucayali in the Peruvian Amazon. On the left, the point color represents the proportion of houses positive for *Ae. aegypti,* with white representing absence, and from blue to red representing an increasingly higher proportion of houses positive for the mosquito. On the right, the same sites are colored by the average number of adult *Ae. aegypti* collected per house, again with white representing zero *Ae. aegypti* and from blue to red representing an increasingly higher average number of adult *Ae. aegypti* per house. The stars highlight the three major cities. Maps created in qGIS with shape files from the Peruvian Government [27] and the United Nations Office for the Coordination of Humanitarian Affairs [28].

We observed a wide range of infestation levels (Fig 2; Table 1). Among the large cities, Iquitos had both the highest adult house index (percent of houses with at least one adult *Ae. aegypti;* AHI = 91.7%) and highest mean number of adult *Ae. aegypti* per house (mean ± SD = 7.67 ± 9.46). Yurimaguas had an AHI of 83.3% and mean adult number of 2.29 ± 2.05, while Pucallpa had an AHI of 52.9% and mean adult number of 1.83 ± 3.71.

**Table 1:** Infestation levels of adult *Ae. aegypti* and larval mosquitoes by site and urbanization level.

Site	Pop. Size^ζ^	No. Houses Visited	Adult *Ae. aegypti *House Index (%)	Mean No. Adult *Ae. aegypti* (*± *SD)	No. Houses Larval Survey	Larval Index (%)
Large City						
Iquitos	437,620	48	91.7	7.67 (9.46)	0	NA
Pucallpa	310,750	140	52.9	1.83 (3.71)	138	16.7
Yurimaguas	41,827	24	83.3	2.29 (2.05)	24	33.3
Small City						
Contamana	17,429	76	75.0	5.21 (8.93)	68	32.4
Nauta	19,551	67	88.1	5.16 (5.10)	28	46.4
Requena	22,875	97	79.4	3.76 (6.14)	28	28.6
Tamshiyacu	6,181	57	98.2	11.00 (12.84)	30	63.3
Town						
Bretaña *	1,686	132	21.2	0.74 (2.66)	109	14.7
Flor de Punga	1,763	78	79.2	3.77 (3.74)	72	43.1
Jenaro Herrera	3,596	77	83.1	4.97 (8.20)	26	42.3
Juancito	2,124	100	76.0	3.04 (4.46)	62	41.9
Maypuco	1,248	24	83.3	2.58 (1.98)	24	37.5
Tamanco ☨	1,738	162	40.7	1.56 (3.26)	154	29.9
Tierra Blanca ☨	1,602	112	41.1	1.63 (3.51)	109	36.7
Riverine Village						
Aucayo	587	65	84.6	8.48 (9.79)	29	58.6
Barrio Florida	673	26	84.6	2.38 (2.80)	26	46.2
Canelos *	317	60	23.3	0.72 (1.98)	59	32.2
Huacrachiro *	667	87	62.1	3.15 (4.52)	55	41.8
La Pedrera	531	65	38.5	3.62 (9.83)	63	31.7
Nuevo Paris/ Nueva Alejandría	361	45	0	0	45	0
Santa Rosa de Masisea ☨	413	85	68.2	4.05 (7.48)	83	34.9
Tiruntan ☨	640	99	40.4	1.30 (3.12)	99	32.3
Tres Unidos	507	62	24.2	1.39 (4.36)	61	27.9
Victoria *	858	118	19.5	1.00 (3.30)	29	20.7
Road Village						
1 de Febrero *	183	13	30.8	1.23 (2.80)	13	15.4
13 de Febrero *	670	36	22.2	0.89 (2.05)	24	12.5
Cahuide *	794	40	17.5	0.35 (0.92)	40	5.0
El Dorado *	90	21	14.3	0.19 (0.51)	21	4.8
Nuevo Horizonte *	308	21	9.5	0.14 (0.48)	21	9.5
San Jose *	123	20	10.0	0.15 (0.49)	20	10.0

^**ζ**^Big city data [38] and other site data [39,40].

*first report of *Ae. aegypti* presence in the community.

^☨^first dengue outbreak occurred in the community during the year of collections.

Among the four small cities, Tamshiyacu had the highest adult house index and mean adult number (AHI = 98.2%; mean = 11.0 ± 12.8), even surpassing the infestation levels in Iquitos. The other three small cities had an AHI between 75.0% and 88.1% and mean number of adults between 3.76 and 5.21.

Among the seven towns, the AHI ranged from 21.2% in Bretaña to 83.3% in Maypuco. The town with the lowest mean number of adults was also Bretaña (0.74 ± 2.66) and the town with the highest mean number of adults was Jenaro Herrera (4.97 ± 8.20).

Among all 30 sites, only one did not have *Ae. aegypti* – Nueva Alejandría/Nuevo Paris, two small riverine villages next to one another and connected by road, which we considered as one site. The other nine riverine villages all had *Ae. aegypti*, with different levels of infestations. Some were highly infested, such as Aucayo (AHI = 84.6%; mean = 8.48 ± 9.78) and Santa Rosa de Masisea (AHI = 68.2%; mean = 4.05 ± 7.47), while others had low levels of infestation, such as Victoria (AHI = 19.5%; mean = 1 ± 3.30) and Canelos (AHI = 23.3%; mean = 0.72 ± 1.98).

**Fig 3:**
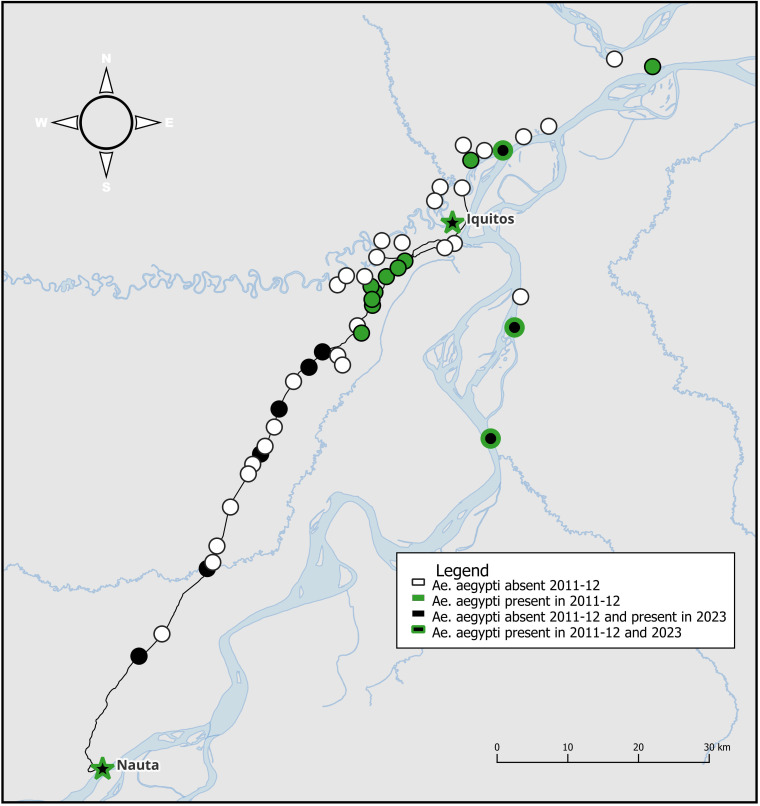
The map shows data extracted from Guagliardo et al. (2014) [21] for the original sampling sites from 2011-2012, conducted within 95 km of Iquitos, overlaid with our data from 2023 for the subset of sites revisited by our team in this region close to the city. White circles indicate locations where *Ae. aegypti* was absent and green where it was present in 2011-12, and where there is no data from 2023. Black circles indicate locations where *Ae. aegypti* was absent in 2011-12 but present in 2023. Black circles with the green outline indicate locations where *Ae. aegypti* was present in both 2011-12 and 2023 collections. Map created in qGIS with shape files from the Peruvian Government [27] and the United Nations Office for the Coordination of Humanitarian Affairs [28].

All six road villages had *Ae. aegypti* infestations, but at relatively low levels, with the AHI ranging from 10.0% in Nuevo Horizonte to 30.8% in 1 de Febrero and the mean number of adult *Ae. aegypti* ranging from 0.14 ± 0.48 to 1.23 ± 2.80, respectively (Fig 3).

*Aedes aegypti* was reported for the first time at ten sites, including four on the river (Bretaña, Canelos, Huacrachiro, and Victoria) and six on the Iquitos-Nauta road (1 de Febrero, 13 de Febrero, Cahuide, El Dorado, Nuevo Horizonte, and San Jose). An additional four communities on the river would have been first reports at the time of site selection, but dengue outbreaks occurred in the months immediately prior to our visit, alerting authorities to the presence of the vector (Tamanco, Tiruntan, Tierra Blanca, and Santa Rosa de Masisea).

### Impact of urbanization level on *Ae. aegypti* infestation levels

#### Presence of *Ae. aegypti.*

**Fig 4:**
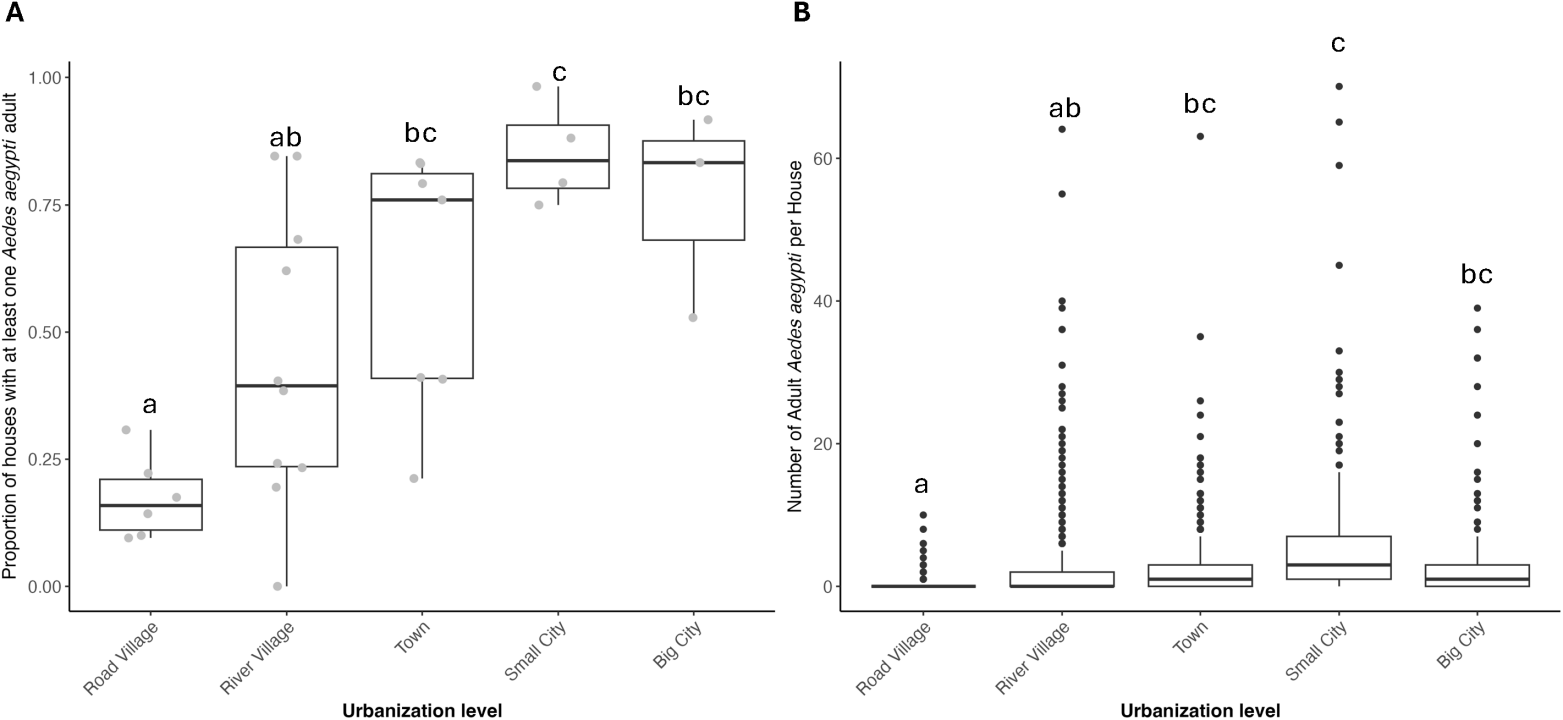
*Aedes aegypti* presence and number by urbanization level. A) The box plot shows the proportion of houses with at least one *Ae. aegypti* adult per site, aggregated by urbanization level. The overlaid gray points display raw data for each site. B) The box plot displays the number of *Ae. aegypti* adults collected per house, aggregated by urbanization level. Notably, there are many outliers, demonstrating that houses at every urbanization level can yield extremely high numbers of adult *Ae. aegypti,* except for road villages, which had fewer and less dramatic outliers. Urbanization levels that do not share a letter in come are significantly different (GLMM, p < 0.05).

Urbanization level had a positive linear relationship (p < 0.0001) and a marginally significant negative quadratic relationship with *Ae. aegypti* adult presence in a house (p = 0.072). In other words, the presence of *Ae. aegypti* adults in houses initially increases with urbanization level, but the quadratic term suggests that the probability of *Ae. aegypti* presence may decline at higher urbanization levels, indicating a more complex, nonlinear pattern. This relationship can be better understood through pairwise comparisons of the estimated marginal means at each urbanization level (Table A in S3 Table). The only significant differences between urbanization levels were lower infestation levels in river villages compared to small cities (p = 0.008) as well as lower infestation levels in road villages compared to the following levels: towns (p = 0.006), small (p < 0.001) and big cities (p = 0.003). The trend can also be visualized in the raw data, which indicates an increase and then plateau of AHI with increasing urbanization (Fig 4A).

#### Number of *Ae. aegypti.*

In the case of the number of *Ae. aegypti* adults per house, the model revealed a positive linear relationship (p < 0.0001) and a negative quadratic relationship with urbanization level (p = 0.028). The pairwise comparisons closely mirrored the household positivity results (Table B in S3 Table), with road villages having significantly lower numbers of *Ae. aegypti* per house compared to towns, small, and big cities (p < 0.010) and river villages with significantly lower numbers compared to small cities (p = 0.027). The trend can also be visualized in the raw data, with an increase in the number of mosquitoes per house with urbanization level until a drop in numbers from small to big cities (Fig 4B).

### Distance from port

**Fig 5:**
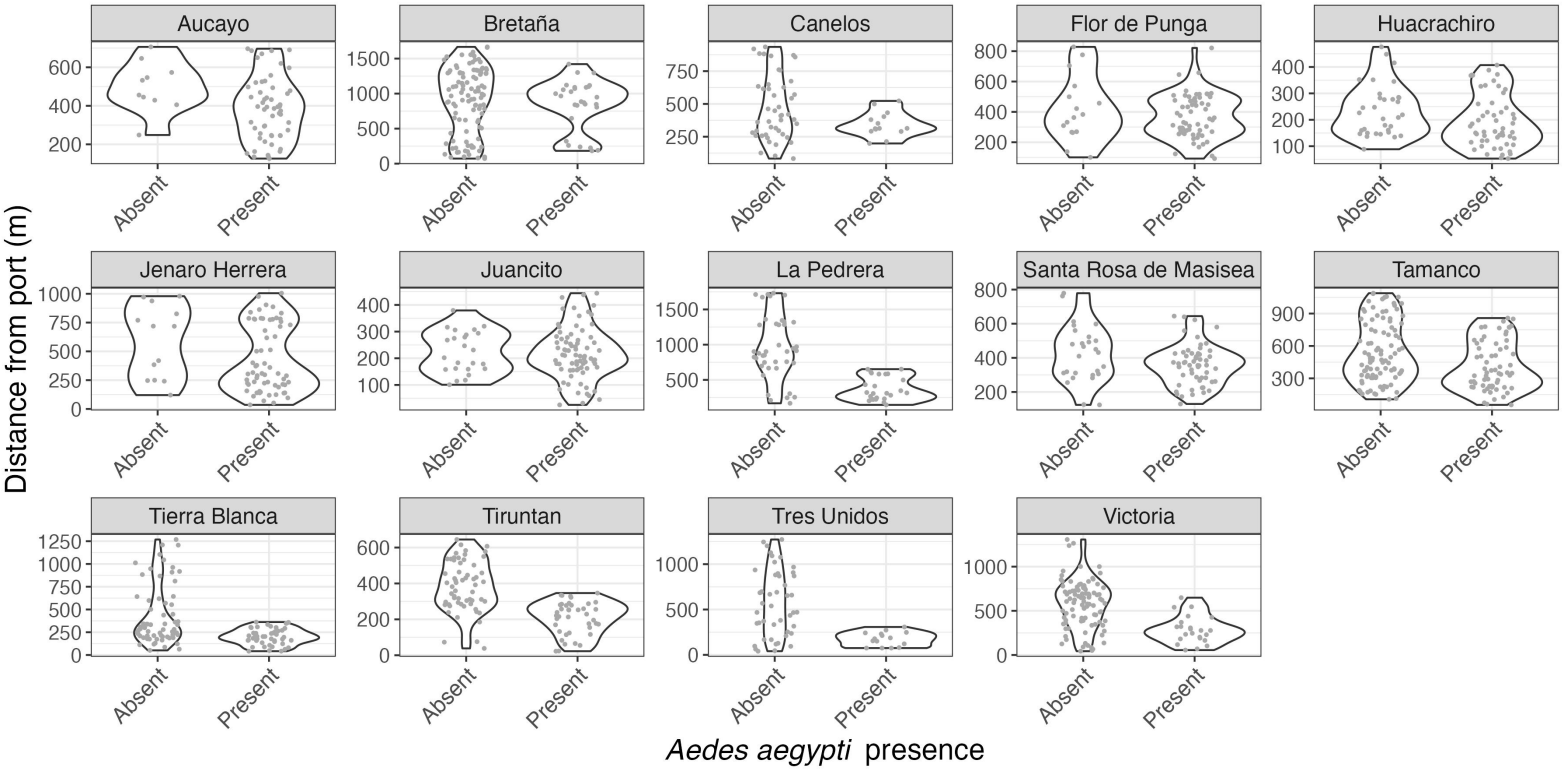
The violin plots display the distance of each household mosquito collection from the community port (m) and whether *Ae. aegypti* was absent or present at the house. Each facet displays the results for a separate riverine village or town, limited to those sites where collections were conducted in transects from the port. The shape of the violin plot demonstrates the density of *Ae. aegypti-*positive and -negative households across the distances sampled. A repeated pattern can be observed: houses without *Ae. aegypti* tended to be concentrated further from the port.

**Fig 6:**
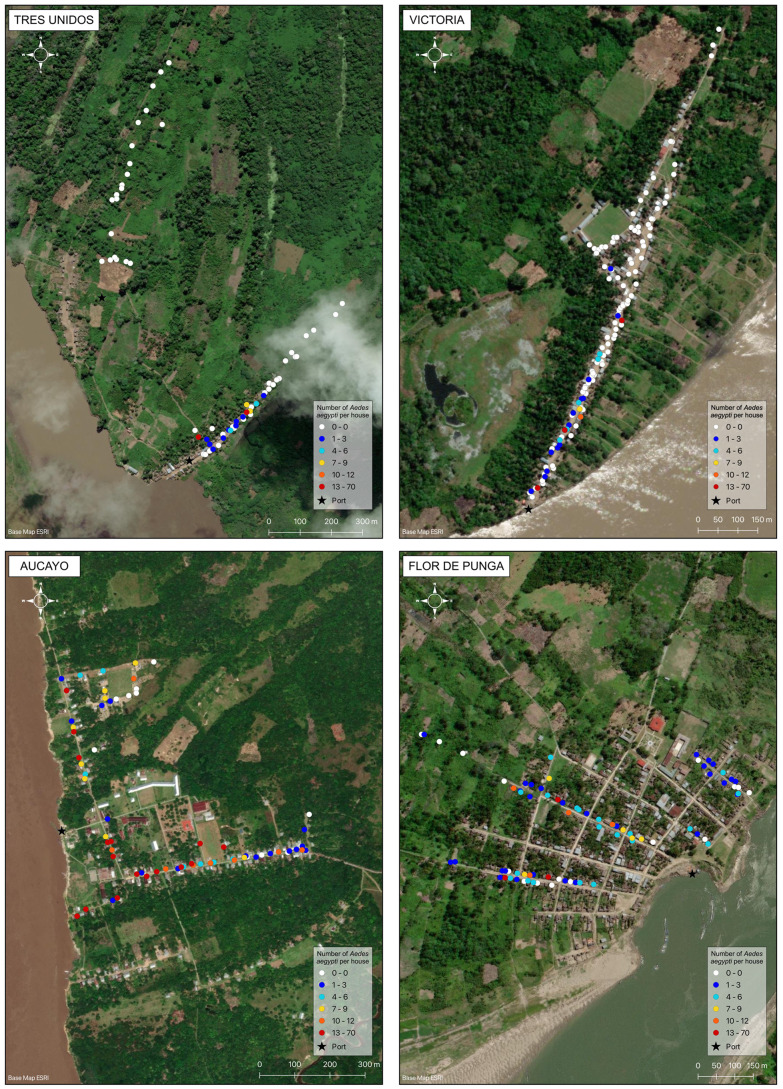
Maps of four sites (3 river villages and 1 town – Flor de Punga). Data is overlaid on ESRI satellite imagery (2023 ESRI) using qGIS. Each point represents a household collection event. The color indicates the number of *Ae. aegypti* collected in the house. White points represent houses where the mosquito was absent and colors from blue to red indicate an increasing number of *Ae. aegypti* per house. In Tres Unidos, the village is divided into two neighborhoods due to erosion resulting in sections of riverbank falling into the river (“desbarrancamiento”), forcing people to move over the past 5 years. The new neighborhood was *Ae. aegypti*-free, while the old neighborhood had *Ae. aegypti* concentrated near the port. Similarly, in Victoria, *Ae. aegypti* was present near the port and disappeared at a certain point into the town. In contrast, *Ae. aegypti* was highly dispersed in Aucayo, although most of the negative houses were located farthest from the port. In Flor de Punga, *Ae. aegypti* was also dispersed throughout the town, with the negative houses scattered without a clear pattern. Additional community-level maps can be found in S1 Fig.

In the riverine towns and villages where we sampled in transects from the port, we repeatedly found *Ae. aegypti* in the houses closer to the ports more often than in houses further from the port (Figs 5 and 6).

#### *Aedes aegypti* presence.

**Fig 7:**
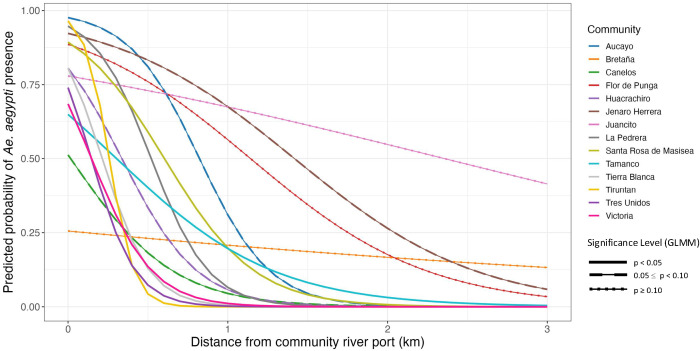
The output of the GLMM supports a repeated relationship between the predicted probability of *Ae. aegypti* presence in a house and the distance of the house from the community port. Each line represents the relationship for each of the 14 communities included in the analysis. Line type represents the significance level of the relationship between presence and distance in each community: solid line indicates p < 0.05 (7 communities), dashed line indicates 0.05 ≤ p < 0.10 (4 communities), and dotted line indicates p ≥ 0.10 (3 communities).

The observed mosquito distribution pattern was interrogated with a GLMM to determine the relationship between distance to port and probability of *Ae. aegypti* presence (Fig 7; Table C in S3 Table). In seven communities, houses closer to the port were more likely to be *Ae. aegypti*-positive than houses further from the port (p < 0.05). Four communities had a marginal relationship between distance and probability of *Ae. aegypti* presence (0.05 ≤ p < 0.10). Three communities had no relationship between distance to port and the likelihood of *Ae. aegypti* presence.

#### *Aedes aegypti* abundance.

A glmmTMB model with a negative binomial distribution (chosen to account for overdispersion of the data) was used to determine whether a similar relationship existed between distance from the port and the number of adult mosquitoes in the house. No clear trend emerged (Table D in S3 Table)– nine of fourteen communities had no significant relationship between distance and number of *Ae. aegypti,* three communities had a significant decrease in the number of mosquitoes further from the port, and two had a significant increase in mosquitoes in houses further to the port.

## Discussion

Our study demonstrates that *Ae. aegypti* has invaded rural communities throughout a large region of the Peruvian Amazon, including numerous remote villages with no previous report of this vector. Our results also suggest a mechanism of invasion – adult *Ae. aegypti* flying off boats docked at community ports. The extent and ubiquity of *Ae. aegypti* invasion is alarming and poses a growing public health threat for numerous vector-borne diseases in rural Amazonia.

Our collections show that distance from a major city does not limit *Ae. aegypti* infestation along major shipping routes. The vector was found at the furthest site sampled, Victoria, 266 km from the closest city, Iquitos. The only site where *Ae. aegypti* was absent was a pair of communities near Pucallpa. This widespread dispersal, independent of distance from a major city, differs from the pattern observed in 2011–2012, when the first systematic study of rural *Ae. aegypti* expansion in Peru was conducted in communities within a 95 km radius of Iquitos [21]. At the time, communities with *Ae. aegypti* tended to be closer to Iquitos than *Ae. aegypti-*negative communities. This change may reflect the vector’s expansion to more remote areas since the original study, neutralizing this pattern. Alternatively, the observed pattern may have been an artifact of the specific sites selected, and invasion to sites further from Iquitos may have already occurred at the time of the 2011–2012 study.

Our collections also show a dramatic expansion of *Ae. aegypti* in road village sites along the Iquitos-Nauta highway. All six villages sampled were free of *Ae. aegypti* during collections conducted in 2011–2012. By 2023, all six communities were infested with *Ae. aegypti,* spanning the full length of the 95 km highway.

The level of *Ae. aegypti* infestation in the rural villages, towns, and small cities was surprising. We documented ten first reports of the mosquito in rural communities and towns. While there was a trend of increasing infestation levels with increasing urbanization, this relationship was not perfectly linear and appears to be driven by the particularly low infestation levels in road villages, as well as the particularly high infestation levels in small cities. There was a remarkable level of variation in infestation levels between sites within the same site size category. It is also notable that *Ae. aegypti* adult indices observed in our study far exceeded those reported in previous studies carried out in Iquitos, Peru, with mean *Ae. aegypti* collected per house rarely exceeding 1 [41–44]. At some rural sites, the percentage of houses positive for adult *Ae. aegypti* and the average number of *Ae. aegypti* per house surpassed infestation levels in the dengue-endemic large cities, demonstrating that entomological risk levels in some rural areas are sufficient to sustain a dengue outbreak. This is particularly striking because sampling locations in big cities was influenced by health department recommendations regarding where there was high *Ae. aegypti* prevalence, which may have biased the results in cities towards higher infestation levels than would be found through random sampling. The risk of dengue transmission in the rural communities is further supported by the history of reported dengue outbreaks in nine of the towns and villages.

Within rural villages and towns, we repeatedly observed that *Ae. aegypti* adults were significantly more likely to be found in houses closer to the community port compared to houses further from port in seven of fourteen communities, with a marginally significant relationship in four additional communities. This result supports previous research in the region attributing long-distance dispersal to *Ae. aegypti* infestation on boats [23,24]. It also adds a layer of detail – the invasions most likely occur at the adult life stage, not the egg or immature life stages. The higher probability of presence near ports suggests that adult female *Ae. aegypti* fly off boats, oviposit in nearby houses, and subsequent generations advance stepwise into the community, creating a distinct distribution pattern radiating from the port. If invasion occurred in the egg or immature stages, we would expect a more heterogenous mosquito distribution, as infested containers should be equally likely to be brought to any house in the community from the boat. Although we believe the distribution patterns indicate adult-stage invasion, it is not conclusive proof and may have another explanation.

There were a few exceptions to this trend. In Juancito and Flor de Punga, which had no relationship between distance and *Ae. aegypti* presence, the species was widespread throughout the town, with an AHI over 75%. The mosquito likely established earlier at these sites and completed its invasion into the towns before we sampled. Three of the four communities with a marginally significant relationship between *Ae. aegypti* presence and distance to the port also had relatively widespread adult presence (greater than 60% AHI), suggesting that the weakening impact of distance to port may also be due to *Ae. aegypti* reaching establishment. This is further supported by prior evidence that Aucayo village was already infested with *Ae. aegypti* during 2008 and 2012 collections [21]. The only low-AHI communities without a significant relationship between distance and *Ae. aegypti* were Bretaña and Canelos. In Bretaña (AHI of 21.2%), there were two focal areas of presence, near the port and town center. We believe that the cluster near town center may be the only example of egg or immature-stage introduction of *Ae. aegypti* among our sites. Canelos (AHI 23.3%) had a marginal relationship between distance and *Ae. aegypti* presence. The town is slightly set back from the river and the *Ae. aegypti-*positive houses were in the half of town closer to the port, beginning just after the houses nearest to the port and ending about halfway through town. It is unclear whether this invasion occurred via adult or egg/immature life stages.

Given the global invasion of *Ae. aegypti,* there is surprisingly limited information regarding the life stage of invasion. Invasions have been associated with transit routes, but it is often unclear whether the invasion occurred via imported eggs, larvae, or adults [22,45,46]. In the case of the secondary dengue vector, *Ae. albopictus,* there is abundant evidence of the role of eggs and larvae as the invading life stages, and to a lesser extent, adults via passive transport in ground vehicles [47–49]. The evidence for passive dispersal of adult mosquitoes via boat presented by this study is unique in its level of detail and replication across sites.

The community-level distribution patterns also show that *Ae. aegypti* is at varying stages of invasion across different communities. In some locations it is well-established, while in others, it appears to be just beginning its invasion near port. Alternatively, this distribution pattern may suggest a failed invasion, in which the mosquito has not been able to advance further into the community. However, this seems unlikely because, anecdotally, the towns do not have internal expansion barriers. It is also possible that differences between communities was an artifact of collection date and seasonal shifts in weather, since each community was only sampled once during the six-month period. However, we do not believe this to be the case given that collections were conducted in the rainy season, and low and high infestation levels were detected across the sampling period, not segregated by date. The identification of these active invasions provides a rare opportunity to monitor and understand the invasion process in real-time.

[50–52] The widespread presence of *Ae. aegypti* in rural Amazonia has significant public health implications. Over one million people live in rural communities in the Peruvian Amazon [39,40,50–52]. With the arrival of *Ae. aegypti,* these rural communities are newly at risk for dengue, Zika, and chikungunya viruses. Many of the communities are located far from hospitals [25,26], with some study sites over 18 hours away by fast boat, which travels intermittently. Communities along less transited rivers face even greater access challenges. As a result of this inaccessibility, severe dengue cases that require immediate hospital care are evacuated by plane, a time-consuming and expensive effort. Early detection, timely hospitalization, and adequate care are critical for survival of hemorrhagic patients, all of which are limited in rural areas [53]. The expanding threat of dengue in the rural communities could result in a disproportionate burden of severe disease compared to urban areas due to the inaccessibility of care.

The widespread invasion of *Ae. aegypti* to rural Amazonia also increases the risk of sylvatic yellow fever virus (YFV) entering an urban transmission cycle. A large YF outbreak occurred across multiple regions in Peru in 1995 [54,55], and between 2000 and 2014, Peru accounted for 37.4% of reported YF cases in the Americas [56]. Deforestation is hypothesized to increase YF outbreaks by bringing canopy-dwelling *Haemogogus* vectors to ground-level through tree felling, increasing mosquito-human contact [55]. This hypothesis was investigated in an ecological study, which failed to uncover a relationship between human activity (e.g., canopy tree loss) and YF case incidence, but the data may have been too coarse to accurately capture the relationship [56]. Recent Peruvian law changes permit more deforestation in the Amazon [57], which may increase YF transmission to people in remote villages, if deforestation is indeed a driver of human exposure. The presence of *Ae. aegypti* in rural areas increases the YFV amplification risk in rural human populations, thereby also increasing the probability of urban transmission by increasing the number of potential conduits to the city. This shifts the current understanding of the YFV rural transmission cycle as dependent on generalist mosquitoes [58], and may greatly expand amplification risk. A similar risk increase is also likely for other emerging arboviruses, such as Mayaro virus [59].

Our study shows an alarming level of *Ae. aegypti* expansion to rural Amazonia and highlights the need for attention to this new rural health threat. Given the public health risk demonstrated by our findings, we immediately alerted the regional health authorities, which has led to health department efforts to expand *Ae. aegypti* surveillance outside of the cities. However, this expanded surveillance will still be concentrated in larger towns due to limited resources.

There is still much to learn about this invasion process. For our study, we selected sites along the main shipping route; most are relatively large compared to other villages in the region, with a higher degree of fluvial transit connection to the cities. These characteristics may make our sites more likely to be invaded by *Ae. aegypti* than smaller, less connected communities. Future studies should focus on smaller, less connected communities to determine if certain community-level characteristics influence *Ae. aegypti* establishment. There should also be investigation into the transmission of dengue in rural communities. And finally, there is an urgent need to identify strategies to control rural *Ae. aegypti* populations in the region and stop further expansion of the species. Our research suggests that there is still time to intervene before the mosquito becomes fully established in many rural communities in the region.

## Supporting information

S1 Table Site characteristics. Characteristics of each site, including department, population, site size, dengue outbreak history, and collection phase.
(DOCX)

S2 Table Generalized linear mixed model details. This table details the 4 GLMMs used in this study, including response variable, family, fixed and random effects, number of sites, number of observations, and other notes.
(DOCX)

S3 Table Emmeans and emtrends results for GLMMs. Four tables reporting the pairwise comparison and trends results for the four models conducted in this study.
(DOCX)

S1 Text Community engagement. This document reports the methods used to conduct community outreach and involve community members in the scientific process.
(DOCX)

S2 Text Larval habitat survey methods and results. This document reports the methods used to collect and analyze data regarding larval habitats, as well as the results and discussion of the findings.
(DOCX)

S3 Text Spanish translation//Traducción a Español. This document provides a translation of the paper to Spanish to improve accessibility//Este documento proporciona una traducción del artículo al español para mejorar la accesibilidad.
(DOCX)

S1 Fig Maps of sites and collection data. ESRI satellite imagery (2023 ESRI) of the 30 sites, with data points overlaid in qGIS depicting the number of adult Ae. aegypti captured during each household collection event.
(PDF)
