# Supplementary material for: Changing dynamics of Aedes aegypti invasion and vector-borne disease risk for rural communities in the Peruvian Amazon
Source: PLoS Negl Trop Dis. 2025 Aug 28;19(8):e0012506. doi: 10.1371/journal.pntd.0012506 (PMC12393723; doi:10.1371/journal.pntd.0012506)
Supplement: S1 Table — Characteristics of each site, including department, population, site size, dengue outbreak history, and collection phase. (DOCX) [file pntd.0012506.s001.docx]

**S1 Table.** Site characteristics

| **Site** | **Department** | **Population*** | **Site Size** | **Dengue Outbreak History** | **Collection Phase** |
| --- | --- | --- | --- | --- | --- |
| Iquitos | Loreto | 144463 | Big City | multiple outbreaks | 1 |
| Barrio Florido | Loreto | 673 | River Village | no outbreak | 2 |
| Aucayo | Loreto | 587 | River Village | no outbreak | 1 |
| Tamshiyacu | Loreto | 6181 | Small City | multiple outbreaks | 1 |
| Nauta | Loreto | 19551 | Small City | multiple outbreaks | 1 |
| Jenaro Herrera | Loreto | 3596 | Town | multiple outbreaks | 1 |
| Requena | Loreto | 22875 | Small City | multiple outbreaks | 1 |
| Flor de Punga | Loreto | 1763 | Town | 1 outbreak | 1 |
| Tamanco | Loreto | 1738 | Town | 1 outbreak | 1 |
| La Pedrera | Loreto | 531 | River Village | 1 outbreak | 1 |
| Tres Unidos | Loreto | 507 | River Village | no outbreak | 1 |
| Bretaña | Loreto | 1686 | Town | no outbreak | 1 |
| Huacrachiro | Loreto | 667 | River Village | no outbreak | 1 |
| Victoria | Loreto | 858 | River Village | no outbreak | 1 |
| Juancito | Loreto | 2124 | Town | multiple outbreaks | 1 |
| Tierra Blanca | Loreto | 1602 | Town | 1 outbreak | 1 |
| Canelos | Loreto | 317 | River Village | no outbreak | 1 |
| Contamana | Loreto | 17429 | Small City | multiple outbreaks | 1 |
| Tiruntan | Loreto | 640 | River Village | 1 outbreak | 1 |
| Maypuco | Loreto | 1248 | Town | 1 outbreak | 2 |
| Yurimaguas | Loreto | 62903 | Big City | multiple outbreaks | 2 |
| 1 de Febrero | Loreto | 183 | Road Village | no outbreak | 2 |
| 13 de Febrero | Loreto | 670 | Road Village | no outbreak | 2 |
| Cahuide | Loreto | 794 | Road Village | no outbreak | 2 |
| El Dorado | Loreto | 90 | Road Village | no outbreak | 2 |
| Nuevo Horizonte | Loreto | 308 | Road Village | no outbreak | 2 |
| San Jose | Loreto | 123 | Road Village | no outbreak | 2 |
| Nuevo Paris/Nueva Alejandria | Ucayali | 361 | River Village | no outbreak | 1 |
| Pucallpa | Ucayali | 144207 | Big City | multiple outbreaks | 1 |
| Santa Rosa de Masisea | Ucayali | 413 | River Village | 1 outbreak | 1 |
| *Populations reflect the 2017 census reports. In the case of big cities, the number reported only includes the population of the city proper and does not include the periphery populations, which are often considered to be part of the city. E.g. the full 2017 Iquitos population was thought to be about 447,000 people (www.macrotrends.net). | | | | | |
