# Supplementary material for: Changing dynamics of Aedes aegypti invasion and vector-borne disease risk for rural communities in the Peruvian Amazon
Source: PLoS Negl Trop Dis. 2025 Aug 28;19(8):e0012506. doi: 10.1371/journal.pntd.0012506 (PMC12393723; doi:10.1371/journal.pntd.0012506)
Supplement: S2 Table — This table details the 4 GLMMs used in this study, including response variable, family, fixed and random effects, number of sites, number of observations, and other notes. (DOCX) [file pntd.0012506.s002.docx]

**S2 Table**: Generalized Linear Mixed Model Details

| Urbanization Level Models | | | | | | |
| --- | --- | --- | --- | --- | --- | --- |
| **Response Variable** | **Family** | **Fixed Effects** | **Random Effects** | **Number of Sites** | **Number of Observations** | **Notes** |
| Presence/ Absence of *Ae. aegypti* | Binomial | Urbanization Level | Site, Collector | 30 | 2055 |  |
| Number of *Ae. aegypti* | Negative Binomial (nbinom1) | Urbanization Level | Site, Collector | 30 | 2055 | Due to overdispersion of the data, a glmmTMB model with a negative binomial distribution was used |
| Distance to Port Models | | | | | | |
| **Response Variable** | **Family** | **Fixed Effects** | **Random Effects** | **Number of Sites** | **Number of Observations** | **Notes** |
| Presence/ Absence of *Ae. aegypti* | Binomial | Site, Distance to port, Interaction between Distance and Site | Collector | 14 | 1301 | This model only considers a subset of the data: the 14 riverine communities with a minimum of 60 data points, where at least 10% of houses were sampled, and where transects were conducted leading away from the port. |
| Number of *Ae. aegypti* | Negative Binomial (with quadratic parameterization; nbinom2) | Site, Distance to port, Interaction between Distance and Site | Collector | 14 | 1301 | Due to overdispersion of the data, a glmmTMB model with a negative binomial distribution was used |
