# Supplementary material for: Changing dynamics of Aedes aegypti invasion and vector-borne disease risk for rural communities in the Peruvian Amazon
Source: PLoS Negl Trop Dis. 2025 Aug 28;19(8):e0012506. doi: 10.1371/journal.pntd.0012506 (PMC12393723; doi:10.1371/journal.pntd.0012506)
Supplement: S3 Table — Four tables reporting the pairwise comparison and trends results for the four models conducted in this study. (DOCX) [file pntd.0012506.s003.docx]

**S3 Table. Emmeans and Emtrends Results for GLMMs**

**Table A. Binomial Urbanization Model Pairwise Comparison Results**: Log Odds of *Ae. aegypti* presence between urbanization levels

| **Contrast** | **Log Odds Ratio** | **SE** | **P value** |
| --- | --- | --- | --- |
| Road Village vs. River Village | 0.230 | 0.145 | 0.199 |
| Road Village vs. Town | 0.100 | 0.067 | 0.006 |
| Road Village vs. Small City | 0.022 | 0.017 | <0.001 |
| Road Village vs. Big City | 0.045 | 0.038 | 0.003 |
| River Village vs. Town | 0.436 | 0.246 | 1.000 |
| River Village vs. Small City | 0.096 | 0.067 | 0.008 |
| River Village vs. Big City | 0.195 | 0.151 | 0.348 |
| Town vs. Small City | 0.221 | 0.161 | 0.385 |
| Town vs. Big City | 0.447 | 0.361 | 1.000 |
| Small City vs. Big City | 2.028 | 1.829 | 1.000 |

*Confidence level: 0.95; Bonferroni method for 10 estimates*

**Table B. Negative Binomial Urbanization Model Pairwise Comparison Results**: Ratio of number of *Ae. aegypti* adults between urbanization levels

| **Urbanization Contrast** | **Ratio** | **SE** | **P value** |
| --- | --- | --- | --- |
| Road Village vs. River Village | 0.311 | 0.133 | 0.064 |
| Road Village vs. Town | 0.200 | 0.090 | 0.004 |
| Road Village vs. Small City | 0.087 | 0.044 | <0.001 |
| Road Village vs. Big City | 0.136 | 0.074 | 0.003 |
| River Village vs. Town | 0.642 | 0.230 | 1.000 |
| River Village vs. Small City | 0.279 | 0.119 | 0.027 |
| River Village vs. Big City | 0.437 | 0.208 | 0.827 |
| Town vs. Small City | 0.434 | 0.194 | 0.616 |
| Town vs. Big City | 0.681 | 0.337 | 1.000 |
| Small City vs. Big City | 1.569 | 0.858 | 1.000 |

*Confidence level: 0.95; Bonferroni method for 10 estimates; Intervals back-transformed from log scale*

**Table C. Binomial Distance to Port Model:** The trend in probability of *Ae. aegypti* presence in a household by distance from port, separated by community

| **Community** | **Distance to Port Trend** | **SE** | **P value** |
| --- | --- | --- | --- |
| Aucayo | -4.51 | 2.341 | 0.054 |
| Bretaña | -0.27 | 0.480 | 0.574 |
| Canelos | -3.106 | 1.770 | 0.079 |
| Flor de Punga | -1.798 | 1.737 | 0.301 |
| Huacrachiro | -4.224 | 2.273 | 0.063 |
| Jenaro Herrera | -1.752 | 1.046 | 0.094 |
| Juancito | -0.536 | 2.535 | 0.833 |
| La Pedrera | -5.55 | 1.382 | 0.000 |
| Santa Rosa de Masisea | -3.523 | 1.778 | 0.048 |
| Tamanco | -2.022 | 0.663 | 0.002 |
| Tierra Blanca | -6.656 | 1.957 | 0.001 |
| Tiruntan | -12.833 | 2.824 | <0.001 |
| Tres Unidos | -7.171 | 2.488 | 0.004 |
| Victoria | -5.251 | 1.277 | <0.001 |

*Confidence level used: 0.95*

**Table D. Negative Binomial Distance to Port Model:** The trend in the number of *Ae. aegypti* per household by distance from port, separated by community

| **Community** | **Distance to Port Trend** | **SE** | **P value** |
| --- | --- | --- | --- |
| Aucayo | -2.585 | 1.109 | 0.020 |
| Bretaña | 2.749 | 0.709 | 0.000 |
| Canelos | 9.196 | 4.383 | 0.036 |
| Flor de Punga | -0.414 | 1.260 | 0.742 |
| Huacrachiro | -0.020 | 1.639 | 0.990 |
| Jenaro Herrera | -1.706 | 0.531 | 0.001 |
| Juancito | -0.140 | 1.344 | 0.917 |
| La Pedrera | 0.917 | 1.508 | 0.543 |
| Santa Rosa de Masisea | -3.287 | 1.149 | 0.004 |
| Tamanco | 0.738 | 0.594 | 0.215 |
| Tierra Blanca | -2.307 | 2.129 | 0.279 |
| Tiruntan | -3.829 | 3.010 | 0.203 |
| Tres Unidos | 2.556 | 3.864 | 0.508 |
| Victoria | 2.046 | 1.750 | 0.242 |

*Confidence level used: 0.95*
