# Supplementary material for: Changing dynamics of Aedes aegypti invasion and vector-borne disease risk for rural communities in the Peruvian Amazon
Source: PLoS Negl Trop Dis. 2025 Aug 28;19(8):e0012506. doi: 10.1371/journal.pntd.0012506 (PMC12393723; doi:10.1371/journal.pntd.0012506)
Supplement: S1 Text — This document reports the methods used to conduct community outreach and involve community members in the scientific process. (DOCX) [file pntd.0012506.s004.docx]

**S1 Text. Community Engagement.**

Our team took several steps to engage communities in the research. We obtained permission from local leaders before beginning collections. In cities and larger towns, we met with environmental health leaders from the respective health departments. In villages and smaller towns, we sought permission from elected government officials and from the Apu in communities with traditional indigenous leadership. The collections were conducted on the ancestral and current lands of several indigenous peoples, including the Cocama-Cocamilla, Shipibo-Conibo, and Urarinas peoples. Once we received permissions from the corresponding leadership, we proceeded to ask individual permission from each household. After each household visit, we discussed our findings with household members and explained how to prevent future larval habitats, if we found any.

In fourteen of the towns and villages, we conducted a workshop about *Ae. aegypti* and dengue and shared details about the scientific purpose of our visit. The workshops were hosted for different audiences depending on the circumstances, including the general community, primary or secondary school children, or the health department employees.

We recruited one representative from each of twenty sites with whom we maintained contact, shared results, and provided opportunities to ask questions and give feedback. Seventeen of the communities were visited at the conclusion of the study after the results were analyzed to share results in meetings with the full community, municipality, and/or the health center employees, depending on the circumstances. Detailed results were also shared with the regional health departments.
