# Supplementary material for: Changing dynamics of Aedes aegypti invasion and vector-borne disease risk for rural communities in the Peruvian Amazon
Source: PLoS Negl Trop Dis. 2025 Aug 28;19(8):e0012506. doi: 10.1371/journal.pntd.0012506 (PMC12393723; doi:10.1371/journal.pntd.0012506)
Supplement: S2 Text — This document reports the methods used to collect and analyze data regarding larval habitats, as well as the results and discussion of the findings. (DOCX) [file pntd.0012506.s005.docx]

**S2 Text. Larval habitat survey methods and results.**

**METHODS:**

**Larval habitat survey methods**

Larval surveys were conducted in and outside of a subset of structures where adult collections were performed (average of 78.8% of surveyed structures per community, range between 24 – 100%), except for Iquitos, where no larval inspections were conducted. It was noted whether a property had at least one container with at least 1 live mosquito larva or pupa, regardless of mosquito species. Time constraints prevented conducting more comprehensive larval surveys across all properties and containers.

**Data Analysis of Larval Infestation Levels and Impact of Larval Habitat on Adult Infestation Levels.** The larval house index was calculated (percentage of houses with at least one mosquito larval habitat with live larvae or pupae). Generalized linear mixed models were then used to examine the relationship between the presence of a mosquito larval habitat on a property and the adult *Ae. aegypti* infestation levels. These models were conducted for the subset of data for which larval habitat data was collected (Table 1). To further explore the interaction between larval habitat and urbanization level, the ‘emtrends’ function from the ‘emmeans’ package [32] was used to estimate the effect of larval habitat across different urbanization levels.

Presence of adult *Ae. aegypti*. A GLMM with a binomial distribution was conducted to determine the relationship between the presence of a mosquito larval habitat and the presence of at least one adult *Ae. aegypti* mosquito for each house. The fixed effects included presence of a larval habitat and urbanization level, and the interaction between the two. The random effects included the site nested within the urbanization level and the collector team.

Number of adult *Ae. aegypti.* Another GLMM was performed to determine the relationship between the presence of a mosquito larval habitat and number of adult *Ae. aegypti* per house using a glmmTMB model with a negative binomial distribution, with the same fixed and random effects as above.

| **Table A in S2 Text. Larval Habitat Models** | | | | | | |
| --- | --- | --- | --- | --- | --- | --- |
| **Response Variable** | **Family** | **Fixed Effects** | **Random Effects** | **Number of Sites** | **Number of Observations** | **Notes** |
| Presence/ Absence of *Ae. aegypti* | Binomial | Urbanization Level, Presence of a larval habitat, Interaction between urbanization and larval habitat | Site, Collector | 28 | 1513 |  |
| Number of *Ae. aegypti* | Negative Binomial (nbinom1) | Urbanization Level, Presence of a larval habitat, Interaction between urbanization and larval habitat | Site, Collector | 28 | 1513 | Due to overdispersion of the data, a glmmTMB model with a negative binomial distribution was used |

**RESULTS:**

**Larval Infestation Levels***.* Most communities had a lower percentage of houses positive for mosquito larvae than the percentage positive for adult *Ae. aegypti*, with the exceptions of Canelos, Tres Unidos, and Victoria, which had a higher percentage positive for mosquito larvae, and Nuevo Horizonte and San Jose, which had the same percentage positive for mosquito larvae and adult *Ae. aegypti.* Notably, all these sites have a low burden of *Ae. aegypti* infestation in general (larval index between 9.5% – 32.2% and AHI between 9.5% – 24.2%).

**Impact of Larval Habitat on Adult Infestation Levels**

Presence of adult *Ae. aegypti*. Properties with a larval site were more likely to have at least one adult *Ae. aegypti* in the house, regardless of site size (GLMM, p < 0.01; Table 2). This pattern is also consistent in the raw data: in big cities, 83.9% of houses with a larval habitat were positive for adult *Ae. aegypti*, whereas 51.1% of houses without a larval habitat were positive for adult *Ae. aegypti.* In small cities, these numbers were 93.4% in houses with larval habitat and 73.9% in houses without larval habitat; towns 71.5% and 39.9%; river villages 74.3% and 32.2%; and road villages 83.3% and 11.0%, respectively.

**Table B in S2 Text.** Binomial Larval Habitat Model Output: Impact of positive larval habitat on presence of adult *Ae. aegypti* by urbanization level

| **Urbanization Level** | **Positive Larval Habitat Trend** | **SE** | **P value** |
| --- | --- | --- | --- |
| Road Village | 3.76 | 0.89 | <0.0001 |
| River Village | 1.93 | 0.23 | <0.0001 |
| Town | 1.28 | 0.22 | <0.0001 |
| Small City | 1.68 | 0.57 | 0.003 |
| Big City | 1.60 | 0.53 | 0.002 |

*Confidence level used: 0.95*

Number of adult *Ae. aegypti.* Similarly, the mean count of adult *Ae. aegypti* per house was higher in houses with a larval habitat compared to houses without a larval habitat (GLMM, p < 0.001; Table 3). Again this pattern is consistent in the raw data: in big cities, houses with a larval habitat had an average of 4.5 ± 6.1 adult *Ae. aegypti* per house, whereas houses without a larval habitat had an average of 1.2 ± 1.8 adults. In small cities, houses with a larval habitat had 8.1 ± 12.2 adults while houses without a larval habitat had 3.5 ± 4.4; in towns 3.8 ± 4.5 and 1.3 ± 3.3, respectively; in river villages, 5.3 ± 8.2 and 1.0 ± 2.8, respectively; and in road villages, 2.6 ± 2.8 and 0.3 ± 0.9, respectively.

**Table C in S2 Text**. Negative Binomial Larval Habitat Model Output: Impact of positive larval habitat on number of adult *Ae. aegypti* per house by urbanization level

| **Urbanization Level** | **Positive Larval Habitat Trend** | **SE** | **P value** |
| --- | --- | --- | --- |
| Road Village | 1.88 | 0.36 | <0.0001 |
| River Village | 1.35 | 0.12 | <0.0001 |
| Town | 0.81 | 0.11 | <0.0001 |
| Small City | 0.62 | 0.16 | 0.0002 |
| Big City | 0.97 | 0.21 | <0.0001 |

*Confidence level used: 0.95*

**DISCUSSION:**

We found that the number of *Ae. aegypti* was significantly higher in houses with a mosquito larval habitat compared to those without, regardless of urbanization level. Similarly, the probability of adult *Ae. aegypti* presence was higher in houses with a larval habitat. These relationships existed despite not identifying mosquito larvae to species. Based off a proportion raised to adulthood and *in-situ* morphological identification of larvae by eye, most larvae were likely *Ae. aegypti.* Furthermore, the presence of a larval habitat, regardless of species, suggests that the container management of the household is permissive of mosquito breeding. The strength of these two relationships indicates that, in this context, the personal management of a household’s containers has a significant influence on that household’s exposure to adult *Ae. aegypti.* This highlights the importance of public health communication to encourage larval habitat removal. However, many houses without larval habitats still had adult *Ae. aegypti,* indicating that individual actions are not sufficient and must be supplemented by community-wide efforts. This aligns with findings of previous studies that reported weak or non-existent relationships between larval indices and adult indices, suggesting that larval indices are not the best predictor of adult *Ae. aegypti* exposure when measured in more detail [1-3].

**REFERENCES:**

1. Parra MCP, Lorenz C, Dibo MR, de Aguiar Milhim BHG, Guirado MM, Nogueira ML, et al. Association between densities of adult and immature stages of *Aedes aegypti* mosquitoes in space and time: implications for vector surveillance. Parasites & Vectors. 2022;15(1):133.

2. Getis A, Morrison AC, Gray K, Scott TW. Characteristics of the spatial pattern of the dengue vector, *Aedes aegypti*, in Iquitos, Peru. Perspectives on Spatial Data Analysis. 2010:203-25.

3. Manrique‐Saide P, Coleman P, McCall P, Lenhart A, Vázquez‐Prokopec G, Davies C. Multi‐scale analysis of the associations among egg, larval and pupal surveys and the presence and abundance of adult female *Aedes aegypti* (Stegomyia aegypti) in the city of Merida, Mexico. Medical and veterinary entomology. 2014;28(3):264-72.
