# Supplementary material for: Changing dynamics of Aedes aegypti invasion and vector-borne disease risk for rural communities in the Peruvian Amazon
Source: PLoS Negl Trop Dis. 2025 Aug 28;19(8):e0012506. doi: 10.1371/journal.pntd.0012506 (PMC12393723; doi:10.1371/journal.pntd.0012506)
Supplement: S3 Text — This document provides a translation of the paper to Spanish to improve accessibility//Este documento proporciona una traducción del artículo al español para mejorar la accesibilidad. (DOCX) [file pntd.0012506.s006.docx]

**S3 Text. Spanish Translation. *Cambios en la dinámica de invasión de* Aedes aegypti *y riesgo de enfermedades transmitidas por vectores en comunidades rurales de la Amazonía peruana***

Kara Fikrig^1^, Arnold O. Noriega^2^, Rosa A. Rodriguez^2^, John Bardales^2^, José Rivas^2^, Becker Reyna^3^, Guido Izquierdo^3^, Gissella M. Vasquez^4^, Ryan T. Larson^4^*, Amy C. Morrison^3,5^, and Laura C. Harrington^1^

^1^Cornell University, Ithaca, New York, USA; ^2^Asociación Benéfica Prisma, Lima, Perú; ^3^Gerencia Regional de Salud, Loreto, Perú; ^4^U.S. Naval Medical Research Unit SOUTH, Lima, Perú; ^5^University of California Davis, Davis, California, USA

*Afiliación actual: Navy and Marine Corps Force Health Protection Command, VA, USA

**Resumen:** Aedes aegypti, el principal vector del virus del dengue, se considera predominantemente un mosquito urbano, especialmente en el continente americano, donde su reemergencia comenzó en las ciudades después del fin de las campañas de erradicación a nivel continental. Los resultados de nuestro estudio divergen de esta narrativa, demostrando la reciente y generalizada invasión rural de Ae. aegypti a lo largo de las principales rutas de navegación en el norte de la Amazonía peruana entre las principales ciudades de Iquitos, Pucallpa y Yurimaguas. Usando aspiradores Prokopack para realizar colectas de mosquitos en interiores, identificamos poblaciones de Ae. aegypti en 29 de los 30 sitios muestreados a lo largo de un gradiente rural-urbano y cuantificamos métricas de adultos de Ae. aegypti. En múltiples casos, los índices de adultos de Ae. aegypti en comunidades rurales fueron iguales o superiores a los índices en ciudades endémicas de dengue, lo que sugiere que el nivel de riesgo entomológico en algunas áreas rurales es suficiente para facilitar la transmisión del dengue. Se muestrearon catorce sitios rurales en transectos desde el puerto fluvial comunitario hacia el punto extremo del pueblo. En siete de estos sitios, las casas más cercanas al puerto tenían significativamente más probabilidades de estar infestadas con adultos de Ae. aegypti que las casas más alejadas de los puertos, y cuatro sitios adicionales mostraron una tendencia similar. Este patrón sugiere que Ae. aegypti todavía está invadiendo activamente muchos sitios rurales, mediante adultos que desembarcan de los barcos en el puerto, encuentran sitios de oviposición cercanos y avanzan paso a paso más adentro del pueblo, con secciones del pueblo aún libres de *Ae. aegypti*. Solo un sitio mostró una señal de invasión a través de la etapa de huevo o larva, con un foco de Ae. aegypti lejos del puerto. La infestación generalizada de Ae. aegypti en áreas rurales es una gran amenaza para la salud pública dada la gran distancia de las comunidades a la atención hospitalaria. Es importante implementar medidas de control ahora antes de que el mosquito se establezca más en zonas de invasión activa.

**Resumen del autor:** El virus del dengue y su vector, el mosquito Aedes aegypti, han sido históricamente considerados un problema urbano en Sudamérica. Desde su reemergencia tras la campaña de erradicación a nivel continental a mediados del siglo 1900, Ae. aegypti ha infestado predominantemente áreas urbanas, dejando a las zonas rurales relativamente libres de los arbovirus que transmite. Este estudio aporta evidencia de que este paradigma está cambiando en la Amazonía peruana. Identificamos infestaciones de Ae. aegypti en 29 de 30 sitios que representan un espectro de urbanización, desde caseríos hasta ciudades. Las métricas de infestación (proporción de casas infestadas y número promedio de adultos de Ae. aegypti por casa) fueron más altas en algunos caseríos que en ciertas ciudades. Observamos un patrón repetido de poblaciones de Ae. aegypti agrupadas cerca de los puertos de los caseríos, lo que sugiere que las invasiones ocurren con frecuencia cuando mosquitos adultos vuelan desde los botes al llegar al puerto, y generaciones subsecuentes se dispersan casa por casa hacia el interior desde el punto de entrada. Este patrón también indica que algunas comunidades rurales están experimentando invasiones activos y que las infestaciones rurales de Ae. aegypti probablemente seguirán expandiéndose, con las poblaciones recién invadidas se establezcan por completo. Este cambio en la ecología de Ae. aegypti en la Amazonía peruana tendrá graves implicancias para la salud pública, aumentando el riesgo de dengue y otros arbovirus para caseríos remotos con acceso limitado a la atención médica.

**Introducción:** Los mosquitos Aedes aegypti transmiten numerosos virus que afectan la salud humana, incluido el virus del dengue, en los trópicos y subtrópicos globales. El virus del dengue causa alrededor de 400 millones de infecciones al año, resultando en más de 40,000 muertes [

1, 2]. La enfermedad puede manifestar un gran rango de morbilidad, incluyendo la necesidad de atención hospitalaria avanzada en la UCI, lo que lleva a un costo acumulado de aproximadamente US$8.9 mil millones [1, 3].

El dengue se reportó por primera vez en Perú en 1990, siguiendo la reemergencia de Ae. aegypti en el país. El mosquito fue declarado erradicado en Perú en 1958, como resultado de la campaña de erradicación de la fiebre amarilla a nivel continental [4, 5]. En 1984, Ae. aegypti fue detectado nuevamente en Iquitos, una ciudad amazónica accesible solo por barco y avión [5]. En el lapso de tres años, Ae. aegypti nuevamente invadió la ciudad de forma rápida, aumentando los niveles de infestación del 1% al 26% entre 1984 y 1988 [5]. El primer brote de dengue reportado en Iquitos ocurrió dos años después [5]. Patrones similares de erradicación, reinfestación, dispersión aumentada y transmisión del dengue por Ae. aegypti se repitieron en gran parte de las Américas durante el mismo período [6].

Debido a que la reinfestación de Ae. aegypti y la transmisión del dengue en las Américas comenzaron en áreas urbanas densamente pobladas, el vector y el virus han sido considerados un problema urbano [7]. Recientemente, el perfil geográfico de la distribución de Ae. aegypti y la transmisión del dengue en las Américas ha comenzado a cambiar, expandiéndose a comunidades rurales más remotas [8]. Ha habido un aumento en los informes de poblaciones rurales de Ae. aegypti en numerosos países del continente [9-13], lo que probablemente se expandirá aún más con el aumento de la conectividad del transporte, el aumento del uso de plásticos y otros desechos que pueden servir como hábitat larval en áreas rurales, y la falta persistente de acceso a agua corriente, lo que obliga a la recolección de agua [8]. Esta tendencia ha recibido poca atención a pesar de los aumentos paralelos en la transmisión del dengue rural, con evidencia de alta seroprevalencia y la circulación de múltiples serotipos en comunidades rurales, particularmente en Colombia y Ecuador [14-17]. Es importante destacar que los registros de esta expansión rural del mosquito y el virus probablemente subestimen su verdadera distribución rural debido a la limitada vigilancia vectorial y de enfermedades en áreas rurales.

La Amazonía peruana del norte es un escenario interesante para explorar la expansión de Ae. aegypti, dada la heterogeneidad de la urbanización y la conectividad fluvial en toda la región. Iquitos, la ciudad donde se identificó por primera vez el dengue en Perú, está conectada por río a las ciudades portuarias de Pucallpa y Yurimaguas, que también tienen transmisión endémica de dengue y poblaciones establecidas de Ae. aegypti [18-20]. Los ríos que conectan estas ciudades sirven como una carretera fluvial para transportar bienes y personas entre las ciudades, así como los cientos de comunidades rurales dispersas a lo largo de los ríos.

Los barcos que navegan por estos ríos son una parte crítica de la dispersión de mosquitos Ae. aegypti a comunidades remotas [21, 22]. Una encuesta de vehículos terrestres y fluviales mostró que todas las formas de tránsito fluvial podrían estar infestadas con Ae. aegypti, con la tasa de infestación más alta en lanchas grandes (71.9%) [23]. Incluso se encontró que las lanchas sostenían la oviposición activa de Ae. aegypti, facilitando el mosquito completar su ciclo de vida a bordo de la lancha [24].

La primera detección de Ae. aegypti en comunidades rurales fuera de Iquitos fue en 2008, a través de un estudio epidemiológico sobre arbovirus realizado por la U.S. Naval Medical Research Unit SOUTH (conocido en aquel momento como Navy Medical Research Center Detachment, NMRCD) y actividades independientes de vigilancia rutinaria realizadas por el Ministerio de Salud del Perú [21]. En 2011-2012, se realizó una encuesta más completa de Ae. aegypti en 34 sitios a lo largo del río Amazonas y los 95 km de carretera entre Iquitos y Nauta (una a ciudad pequeña cerca de Iquitos) [21]. Estas colectas mostraron distribuciones heterogéneas de mosquitos en comunidades ribereñas y patrones espaciales a lo largo de las carreteras. La mitad de las comunidades ribereñas encuestadas estaban infestadas. Las comunidades con un mayor tamaño de población humana y una distancia más cercana a Iquitos tenían más probabilidades de estar infestadas con Ae. aegypti. Las colectas a lo largo de la carretera mostraron presencia de Ae. aegypti en todas las comunidades hasta un punto discreto, después del cual todas las comunidades posteriores fueron negativas. En ese momento, el punto más lejano de expansión de Ae. aegypti desde Iquitos era de 37.1 km por río y 19.3 km por carretera [21].

Desde entonces, no ha habido una caracterización sistemática de las poblaciones rurales de Ae. aegypti en la Amazonía peruana, ni una caracterización del mosquito más allá de 95 km de Iquitos. La falta de información sobre el movimiento y presencia del vector en áreas remotas es particularmente preocupante porque las comunidades en estas áreas están ubicadas lejos de las instalaciones médicas y del cuidado necesario para tratar el dengue severo. La combinación de recursos insuficientes y la ubicación remota ha creado un acceso altamente inequitativo en la atención médica para las comunidades rurales en la Amazonía peruana [25, 26].

Esta información ecológica básica sobre la distribución de Ae. aegypti es vital para comprender el riesgo de dengue rural en esta región. Para generar esta información, realizamos colectas de mosquitos en treinta sitios distribuidos dentro de una vasta extensión de la Amazonía peruana del norte, a lo largo de más de 1,000 km de río, a través de un gradiente rural-urbano, para determinar la distribución de Ae. aegypti y medir el riesgo entomológico relativo para la transmisión de virus transmitidos por Aedes en la región. Teníamos la expectativa de observar una tendencia en los niveles de infestación asociados con el grado de urbanización, anticipando que las comunidades más pobladas tendrían más probabilidades de estar infestadas y tener índices entomológicos más altos. De hecho, detectamos una tendencia así, pero fue más débil de lo esperado debido a la presencia casi ubicuidad de Ae. aegypti a lo largo del transecto. Detectamos infestaciones de Ae. aegypti en casi todos los sitios encuestados y medimos altos índices entomológicos en numerosas pequeñas comunidades rurales, lo que indica que el proceso de expansión está más avanzado de lo previamente imaginado.

**Métodos**

En las siguientes secciones, describimos el razonamiento que utilizamos para seleccionar nuestros sitios, seguido de las características de los sitios seleccionados, métodos de colecta de mosquitos, detalles de datos geográficos y métodos de análisis estadístico. Luego, terminamos con declaraciones sobre la disponibilidad de datos, permisos y la disponibilidad de una traducción al español de este documento.

***Selección de sitios*:** En este estudio, buscamos caracterizar la invasión de Ae. aegypti en comunidades remotas a lo largo de un gradiente rural-urbano en los departamentos Loreto y Ucayali en norte de la Amazonía peruana. Esta región de la Amazonía tiene varias ciudades grandes y pequeñas, y muchos pueblos y caseríos dispersos a lo largo de la inmensa extensión de selva tropical. Las comunidades están principalmente conectadas por la compleja matriz de ríos que atraviesan el bosque. La colecta de mosquitos se concentró a lo largo del sistema fluvial que conecta Iquitos con Pucallpa, una de las dos ciudades portuarias que suministran a Iquitos bienes desde fuera de la Amazonía (Fig. 1), elevando la probabilidad de dispersión a larga distancia de Ae. aegypti facilitada por las lanchas.

Nuestro objetivo era seleccionar sitios a lo largo de gradientes del tamaño de la población e historial de brotes de dengue confirmados (ninguno, un brote o múltiples brotes) y distribuidos geográficamente a lo largo del transecto fluvial. Utilizamos registros del departamento de salud de Loreto sobre campañas de fumigación con insecticidas dirigidas por el gobierno en comunidades durante los cinco años anteriores, donde un registro se consideraba sinónimo de un brote de dengue o de transmisión de malaria. La malaria no es endémica en las comunidades seleccionadas, por lo que el tratamiento con insecticidas fue en respuesta a brotes de dengue.. Donde no había registro de uso de insecticidas, no había un registro previo de la presencia de Ae. aegypti. La selección final de los sitios se basó en una síntesis del censo, la ubicación geográfica y los datos de uso de insecticidas del departamento de salud; sin embargo, dado el gran número de comunidades rurales, la lista final de sitios fue determinada en gran parte por consideraciones logísticas: los mismos criterios de selección podrían haber resultado en un conjunto diferente de sitios a lo largo del mismo transecto.

Las colectas se realizaron en dos fases. La Fase 1 involucró colectas más exhaustivas, con un promedio de 87.24 viviendas por sitio (rango: 45–162), en comparación con 25 viviendas por sitio (rango: 13–40) en la Fase 2. Se seleccionaron dieciocho sitios entre Iquitos y Pucallpa y un sitio al otro lado de Pucallpa, distribuidos de manera aproximadamente equidistante a lo largo del transecto, además de las dos ciudades. La Fase 2 se inició después de la Fase 1 para aprovechar un excedente de tiempo en campo, lo que permitió visitas breves a 9 sitios adicionales que no estaban planificados inicialmente. Las colectas de la Fase 2 fueron más cortas e incluyeron menos viviendas por sitio debido a limitaciones logísticas, pero todos los demás métodos se mantuvieron consistentes. Las colectas en la Fase 2 incluyeron 6 sitios a lo largo del corto tramo de carretera que conecta Iquitos con la pequeña ciudad de Nauta, todos previamente negativos para Ae. aegypti en las colectas de 2011-12 realizadas por Guagliardo et al [21], así como un sitio en el río Amazonas al norte de Iquitos, una comunidad en la ruta fluvial del río Marañón hacia la otra ciudad portuaria, Yurimaguas, y la ciudad de Yurimaguas misma. En total, se recolectaron muestras en 30 sitios, con 21 sitios en la Fase 1 y 9 sitios en la Fase 2.

**Figura 1.** Mapa de sitios de muestreo (30) en los departamentos de Loreto y Ucayali, demostrando el tamaño del sitio en símbolos (negro en la leyenda) y el historial de brotes de dengue en color (cuadrado en la leyenda). Mapa creado en qGIS con archivos shapefile del Gobierno del Perú [27] y de la United Nations Office for Coordination of Hunanitarian Affairs (OCHA) [28].

***Características del sitio*:** Los sitios están geográficamente dispersos a lo largo de dos rutas principales de tránsito fluvial: Iquitos - Pucallpa e Iquitos - Yurimaguas. La ruta Iquitos - Pucallpa abarca una distancia euclidiana de 536 km, lo que equivalente a 1,068 km y más de 36 horas de viaje en los botes rápidos (pongueros) que transitan los ríos Ucayali, Puinahua y Amazonas. La ruta Iquitos - Yurimaguas abarca una distancia euclidiana de 399 km, lo que es equivalente a 661 km y más de 20 horas de viaje en los botes rápidos a lo largo de los ríos Huallaga y Marañón. También muestreamos dos comunidades más allá de las ciudades a lo largo de estos ríos. El rango de población de los sitios es de 120 a 484,000 personas. Tres sitios se consideran ciudades grandes (>23,000 personas), cuatro son ciudades pequeñas (5,000 - 23,000 personas), siete son pueblos (1,000 a 4,999 personas) y dieciséis son caseríos (<1,000 personas; diez a lo largo de los ríos (= caserío ribereño) y seis a lo largo de la carretera Iquitos-Nauta (= caserío de carretera)).

Entre los veintiún sitios encuestados en la fase 1 a lo largo de la ruta fluvial Iquitos-Pucallpa, siete comunidades nunca reportaron un brote de dengue, seis comunidades reportaron un solo brote de dengue y ocho reportaron múltiples brotes antes del momento de nuestras colectas (incluyendo Iquitos y Pucallpa, consideradas endémicas para el dengue). Cinco sitios con un brote fueron inicialmente seleccionados como sitios sin historial de dengue (y sin conocimiento *a priori* de la presencia de Ae. aegypti), pero cuatro experimentaron un brote de dengue durante los meses entre la selección del sitio y la visita al sitio, y uno experimentó un brote justo más de cinco años antes, fuera del periodo de cinco años de datos que recibimos del departamento de salud. Entre los nueve sitios encuestados en la fase 2, siete comunidades no tenían registro de dengue transmitido localmente ni presencia de Ae. aegypti (todas los caseríos de carretera y el caserío ribereño más allá de Iquitos), uno tenía historial de un solo brote y uno tenía múltiples brotes de dengue (Yurimaguas, también considerada endémica). Consulte la S1 Tabla para ver las características de cada sitio.

Aunque el nivel de urbanización del sitio se clasificó por el tamaño de la población, otras características del sitio están asociadas con estas designaciones. Las caseríos y pueblos tenían electrificación limitada, con electricidad generada durante aproximadamente 3 a 4 horas al día (excepto por un pueblo con electricidad todo el día), mientras que las ciudades tenían electricidad todo el día. Las casas en caseríos y pueblos mayormente estaban hechas de madera, con techos de metal o de hojas de palma. En las ciudades grandes, las casas mayormente estaban hechas de concreto con techos de metal, y las ciudades pequeñas tenían una mezcla de los dos tipos de vivienda. La mayoría de los caseríos y pueblos no tenían ninguna forma de manejo de residuos, excepto algunas capitales de distrito que tenían recolección limitada de residuos y un botadero en el bosque. Las ciudades tenían un manejo de residuos más establecida. Aunque consideramos los caseríos y pueblos en este contexto como rurales, las casas estaban notablemente agrupadas, con tierras agrícolas dispersas a lo largo del río y hacia el bosque. Los patios eran típicamente un poco más grandes que los de las ciudades, pero la densidad de población no es tan diferente como en otras regiones con dicotomías rural/urbana.

***Colecta de Mosquitos:*** Antes de iniciar las colectas de mosquitos, nuestro equipo llevó a cabo extensas actividades de involucramiento comunitario, descritas en detalle en el S1 Texto. Un equipo de cinco colectores (cuatro por sitio) realizó colectas de mosquitos con aspiradores Prokopack [29], operados dentro de las estructuras entre las 07:00 y las 18:00 horas. Las colectas se llevaron a cabo en viviendas, tiendas, oficinas y estructuras de uso mixto (espacios de tienda u oficina junto a espacios habitacionales). Las estructuras fueron revisadas sistemáticamente en todos los espacios a los que se permitió el ingreso de los colectores, utilizando linternas frontales y perturbación física de todas las superficies, incluyendo debajo de los muebles con los aspiradores para recolectar mosquitos volando y reposando. El tiempo de recolección varió según el tamaño y la estructura interna del hogar, pero generalmente entre 5 y 30 minutos.

Los mosquitos se mantuvieron vivos en vasos de recolección de 0.7 L cubiertos con malla, etiquetados con un código único por estructura. Los mosquitos fueron matados al final del día colocando los vasos dentro de una bolsa sellada con acetona durante aproximadamente 15 minutos. Los especímenes fueron clasificados y todos los *Ae. aegypti* fueron separados y confirmados en base a características taxonómicas clave usando un microscopio de campo [30]. Las limitaciones de tiempo y recursos impidieron la identificación de todas las demás especies de mosquitos. Cabe destacar que *Ae. albopictus* no está presente en la Amazonía peruana y no fue detectado en ninguno de los sitios.

También se realizaron encuestas larvales limitadas, cuyos métodos y resultados se reportan en el S2 Texto.

En las colectas de la fase 1 (a lo largo del transecto Iquitos-Pucallpa), el enfoque de muestreo fue diseñado para incluir áreas cerca y lejos de los puertos y se adaptó en función del tamaño de la comunidad y los horarios de los barcos. En los caseríos, se visitó un alto porcentaje de casas (37 - 93% de las casas según los conteos del censo de hogares de 2017 [31, 32]) y la mayoría de las que se acercaron permitieron la entrada a nuestro equipo. En los pueblos, colectamos en áreas cerca y lejos de los puertos comunitarios; siempre cuando fue posible, colectamos en un transecto desde el puerto del pueblo hasta las residencias más alejadas del río. En los pueblos, muestreamos el 12 - 31% de las casas. En las ciudades, también colectamos cerca y lejos de los puertos. Notablemente, las colectas de la ciudad fueron las menos representativas de toda la ciudad. La proporción de casas encuestadas fue baja (< 0.2% de las casas) y el área de muestreo fue influenciada por las recomendaciones del departamento de salud sobre dónde había alta prevalencia de Ae. aegypti.

En la fase dos (comunidades a lo largo de la carretera Iquitos-Nauta, la ruta del río Yurimaguas y río abajo desde Iquitos), las colectas se limitaron a un número menor de casas (13 - 40 casas; cobertura que varía entre el 13 - 70% para los caseríos, el 8 - 13% para los pueblos y el 0.1% para la ciudad, Yurimaguas).

Muestreamos de enero a junio de 2023, con la mayoría de las colecciones correspondientes al período de mayor precipitación, niveles de río y transmisión de dengue. Cada comunidad fue visitada una sola vez durante el período de colecta.

Dependiendo del tamaño de la edificación y otras consideraciones logísticas, las colectas a veces fueron realizadas por un colector, a veces por dos colectores y raramente por tres o cuatro colectores. Cinco colectores diferentes participaron en todo el estudio, pero solo tres o cuatro colectores participaron en las colecciones para cualquier sitio dado.

***Registro de Datos Geográficos*:** En el momento del muestreo, se tomó un punto GPS en la puerta principal de la estructura utilizando la aplicación UTM Geo Map (Y2 Tech, Indonesia). El punto GPS se tomó cuando la precisión informada por la aplicación estaba dentro de 3 m. Las coordenadas GPS se cargaron en qGIS (versión 3.30) y se superpusieron con imágenes satelitales de ESRI de 2023 (2023 ESRI). Se midió la distancia euclidiana entre cada punto de muestreo y el puerto fluvial comunitario (identificado a través de imágenes tomadas durante la temporada de creciente). En algunas comunidades, hay dos puertos separados para el creciente (cuando el río está alto) y la temporada de vaciante (con el rió está bajo), o para lanchas y barcos de pasajeros. En estos casos, seleccionamos la distancia al más cercano de los dos puertos para cada punto de muestreo para los análisis estadísticos.

***Análisis de Datos*:** Todos los análisis se realizaron en R versión 4.3.0 [33]. Un resumen de todos los modelos estadísticos realizados se encuentra en la S2 Tabla.

**Niveles de Infestación de** Aedes aegypti**:** Para cada comunidad, se calcularon las siguientes métricas: índice de adultos (porcentaje de casas infestadas con adultos de Ae. aegypti; AHI) y número promedio de adultos (número promedio de adultos de Ae. aegypti por casa). Los mapas que muestran los niveles de infestación se crearon utilizando qGIS.

**Impacto del nivel de Urbanización en los Niveles de Infestación de** Ae. aegypti**:** Se utilizaron modelos lineales generalizados mixtos (GLMM) para determinar el impacto del nivel de urbanización (el tamaño del sitio) en las métricas de infestación (presencia de adultos de Ae. aegypti y número de adultos de Ae. aegypti por casa). Se realizaron comparaciones por pares post hoc con las funciones 'emmeans' y 'pairs' con ajuste de Bonferroni utilizando el paquete 'emmeans' [34] para comparar los medios marginales estimados de las métricas de infestación para cada nivel de urbanización, ajustando para otras variables en el modelo. La urbanización se consideró una variable categórica ordinal con cinco niveles del tamaño de la población: caserío de carretera, caserío ribereño, pueblo, ciudad pequeña y ciudad grande. Cabe destacar que los caseríos de carretera no son uniformemente más pequeñas que los caseríos ribereños, pero tenían una población más baja en promedio y se agruparon por separado debido a la ecología distintito de sus ubicaciones.

**Presencia de** Ae. aegypti**.** Se realizó un GLMM para determinar el impacto del nivel de urbanización en la probabilidad de que una casa fuera negativa (0) o positiva (1) para al menos un adulto de Ae. aegypti utilizando el paquete lme4 [35] con una distribución binomial. El efecto fijo fue el nivel de urbanización, y los efectos aleatorios incluyeron el sitio y el equipo de colectores (cada equipo de colecta único, ya sea un individuo o una combinación de individuos, se consideró un nivel separado de la variable).

**Número de** Ae. aegypti**.** Se realizó otro GLMM para determinar la relación entre el nivel de urbanización y el número de adultos de Ae. aegypti por casa. Debido a la sobredispersión de los datos, se utilizó un modelo glmmTMB con una distribución binomial negativa [36], con los mismos efectos fijos y aleatorios que antes.

**Impacto de la distancia al puerto en los niveles de infestación.** Se realizaron modelos lineales generalizados mixtos en un subconjunto de comunidades para comprender mejor el impacto de la distancia al puerto comunitario en la infestación de Ae. aegypti. Los análisis se limitaron a comunidades ribereñas con un mínimo de 60 puntos de datos, donde al menos el 10% de las casas fueron muestreadas (basado en el número de hogares en el censo de 2017 [31, 32]) y donde realizamos transectos alejándose del puerto. Este subconjunto incluyó 14 comunidades. Para evaluar cómo varía el efecto de la distancia desde el puerto por comunidad, utilizamos la función 'emtrends' [34] para estimar este efecto para cada comunidad.

**Presencia de** Ae. aegypti**.** Primero, realizamos un GLMM con una distribución binomial para evaluar el impacto de la distancia desde el puerto en si una casa era positiva para al menos un adulto de Ae. aegypti. Los efectos fijos incluyeron el sitio, la distancia (km) de la casa al puerto y la interacción de los dos. El equipo de colectores se incluyó como un efecto aleatorio. Se utilizó el optimizador BOBYQA con 200,000 iteraciones.

**Número de** Ae. aegypti**.** Usamos un GLMM para determinar el impacto de la distancia al puerto en el número de mosquitos adultos por casa, utilizando glmmTMB con una distribución binomial negativa con una parametrización cuadrática (familia nbinom2) para tener en cuenta la sobredispersión de los datos [36]. Usamos los mismos efectos fijos y aleatorios que en el modelo de presencia.

***Declaración de Ética.*** Se obtuvieron todos los permisos y aprobaciones necesarios, incluyendo el permiso de colección de SERFOR (N°000066-2024-MIDAGRI-SERFOR-DGGSPFFS-DGSPFS), la aprobación de la Gerencia Regional de Salud (N° 256-2022-GRL-DRSL/30.09-INVESTIGACIÓN), y las colecciones fueron consideradas exentas por las Juntas de Revisión Institucional del U.S. Naval Medical Research Unit SOUTH (NAMRU6.P0003) y la Universidad de Cornell (IRB0145012). Se obtuvo el consentimiento verbal formal de los líderes de las comunidades, así como de cada hogar involucrado (ver S1 Texto).

***Disponibilidad de Datos.*** Los datos y el código que respaldan los resultados de este estudio están disponibles de forma abierta en el repositorio institucional de la Biblioteca de la Universidad de Cornell, eCommons, en <https://doi.org/10.7298/fynw-8v53> [37].

***Traducción al español.*** Este documento ha sido traducido al español para mejorar la accesibilidad en la región donde se realizó la investigación (ver S3 Texto).

**Resultados**

***Niveles de infestación de*** Ae. aegypti***:*** Observamos un rango amplio de niveles de infestación (Fig. 2; Tabla 1). Entre las ciudades grandes, Iquitos tuvo tanto el índice adultas más alto (porcentaje de casas con al menos un adulto de Ae. aegypti; AHI = 91.7%) como el mayor número promedio de adultos de Ae. aegypti por casa (media ± SD = 7.67 ± 9.46). Yurimaguas tuvo un AHI de 83.3% y promedio adultos de 2.29 ± 2.05, mientras que Pucallpa tuvo un AHI de 52.9% y promedio adulto de 1.83 ± 3.71.

**Figura 2. Mapas de los niveles de infestación de adultos de** Ae. aegypti **en nuestros sitios de muestreo en los departamentos Loreto y Ucayali en la Amazonía peruana.** A la izquierda, el color del punto representa la proporción de casas positivas para Ae. aegypti, con el blanco representando la ausencia, y de azul a rojo representando una proporción cada vez mayor de casas positivas para el mosquito. A la derecha, los mismos sitios están coloreados por el número promedio de adultos de Ae. aegypti recolectados por casa, nuevamente con el blanco representando cero Ae. aegypti y de azul a rojo representando un número promedio cada vez mayor de adultos de Ae. aegypti por casa. Las estrellas destacan las tres ciudades principales. Mapas creados en qGIS con archivos shapefile del Gobierno del Perú [27] y de la United Nations Office for the Coordination of Humanitarian Affairs [28].

Entre las cuatro ciudades pequeñas, Tamshiyacu tuvo el índice adulto más alto y el mayor número promedio de adultos (AHI= 98.2%; media = 11.0 ± 12.8), incluso superando los niveles de infestación en Iquitos. Las otras tres ciudades pequeñas tenían un AHI entre 75.0% y 88.1% y un promedio de adultos entre 3.76 y 5.21.

Entre los siete pueblos, el AHI fue entre 21.2% en Bretaña y 83.3% en Maypuco. El pueblo con el número promedio de adultos más bajo también fue Bretaña (0.74 ± 2.66) y el pueblo con el promedio más alto fue Jenaro Herrera (4.97 ± 8.20).

Entre los 30 sitios, solo uno no tenía Ae. aegypti – Nueva Alejandría/Nuevo Paris, dos caseríos ribereños pequeños, uno al lado del otro y conectados por carretera, que consideramos como un solo sitio. Los otros nueve caseríos ribereños tenían Ae. aegypti, con diferentes niveles de infestación. Algunas estaban altamente infestadas, como Aucayo (AHI= 84.6%; media=8.48 ± 9.78) y Santa Rosa de Masisea (AHI= 68.2%; media=4.05 ± 7.47), mientras que otras tenían niveles bajos de infestación, como Victoria (AHI= 19.5%; media=1 ± 3.30) y Canelos (AHI= 23.3%; media = 0.72 ± 1.98).

Los seis caseríos de carretera tenían infestaciones de Ae. aegypti, pero en niveles relativamente bajos, con el AHI entre 10.0% en Nuevo Horizonte y 30.8% en 1 de febrero y el número promedio de adultos de Ae. aegypti entre 0.14 ± 0.48 y 1.23 ± 2.80, respectivamente (Fig. 3).

**Figura 3.** El mapa muestra datos extraídos de Guagliardo et al. (2014) [21] correspondientes a los sitios de muestreo originales de 2011-2012, realizados dentro de un radio de 95 km de Iquitos, superpuestos con nuestros datos de 2023 para el subconjunto de sitios revisitados por nuestro equipo en esta región cercana a la ciudad. Los círculos blancos indican ubicaciones donde Ae. aegypti estuvo ausente en 2011-12 y donde no hay datos de 2023. Los círculos verdes indican presencia de Ae. aegypti en 2011-12, también sin datos de 2023. Los círculos negros indican ubicaciones donde Ae. aegypti estuvo ausente en 2011-12 pero presente en 2023. Los círculos negros con borde verde indican ubicaciones donde Ae. aegypti estuvo presente tanto en las colectas de 2011-12 como en las de 2023. Mapa creado en qGIS con archivos shapefile del Gobierno del Perú [27] y de la United Nations Office for the Coordination of Humanitarian Affairs [28].

Se reporta la presencia de *Ae. aegypti* por primera vez en diez sitios, incluyendo cuatro por el río (Bretaña, Canelos, Huacrachiro y Victoria) y seis por la carretera Iquitos-Nauta (1 de Febrero, 13 de Febrero, Cahuide, El Dorado, Nuevo Horizonte y San José). Otras cuatro comunidades por el río habrían sido primeros reportes en el momento de la selección de sitios, pero ocurrieron brotes de dengue en los meses inmediatamente previos a nuestra visita, alertando a las autoridades sobre la presencia del vector (Tamanco, Tiruntán, Tierra Blanca y Santa Rosa de Masisea).

*Impacto del Tamaño del Sitio en los Niveles de Infestación de* Ae. aegypti*.*

Presencia de *Ae. aegypti*. El nivel de urbanización tuvo una relación lineal positiva (p<0.0001) y una relación cuadrática negativa marginalmente significativa con la presencia de adultos de *Ae. aegypti* por casa (p=0.072). Es decir que la presencia de adultos de *Ae. aegypti* en las casas inicialmente aumenta con el nivel de urbanización, pero el término cuadrático sugiere que la probabilidad de presencia de Ae. aegypti podría disminuir en niveles más altos de urbanización, lo que indica un patrón más complejo y no lineal. Esta relación se puede entender mejor a través de comparaciones por pares de los medios marginales estimados entre cada nivel de urbanización (Tabla A en S3 Tabla). Las únicas diferencias significativas entre los niveles de urbanización fueron los niveles de infestación más bajos en los caseríos de carretera en comparación con los pueblos (p=0.006), las ciudades pequeñas (p<0.001) y las ciudades grandes (p=0.003), así como niveles de infestación más bajos en los caseríos ribereños en comparación con las ciudades pequeñas (p=0.008). La tendencia también puede visualizarse en la data sin modelar, que indican que el AHÍ aumenta y después nivela con el aumento de la urbanización (Fig. 4A).

Número de *Ae. aegypti*. En el caso del número de adultos de *Ae. aegypti* por casa, el modelo reveló una relación lineal positiva (p<0.0001) y una relación cuadrática negativa con el nivel de urbanización (p=0.028). Las comparaciones por pares fueron muy parecidos a los resultados de AHI (Tabla B en S3 Tabla), con los caseríos de carretera teniendo números de *Ae. aegypti* por casa significativamente más bajos en comparación con los pueblos, las ciudades pequeñas y las ciudades grandes (p<0.010) y los caseríos ribereños con números significativamente más bajos en comparación con las ciudades pequeñas (p=0.027). La tendencia también puede visualizarse en la data sin modelar, con un aumento en el número de mosquitos por casa con el nivel de urbanización hasta una disminución entre las ciudades pequeñas a las grandes (Fig. 4B).

**Figura 4.** A) El diagrama de cajas muestra la proporción de casas con al menos un adulto de Ae. aegypti por sitio, agrupado por nivel de urbanización. Los puntos grises superpuestos muestran la data para cada sitio. B) El diagrama de cajas muestra el número de adultos de Ae. aegypti colectados por casa, agrupado por nivel de urbanización. Es notable que hay muchos valores atípicos, demostrando que las casas en cada nivel de urbanización pueden tener números extremadamente altos de adultos de Ae. aegypti, excepto por los caseríos de carretera, que tuvieron menos atípicos y atípicos menos extremos. Los niveles de urbanización que no comparten una letra en común son significativamente diferentes (GLMM, p < 0.05).

| **Sitio**  **Tabla 1.** Niveles de infestación de adultos de Ae. aegypti y mosquitos larvales por sitio y nivel urbanización | **Población^ζ^** | **No. Casas Vistadas** | **Indice Adulto de *Ae aegypti* (%)** | **No. Promedio de *Ae. aegypti Adulto* (*±* SD)** | **No. Casas Encuesta Larval** | **Indice Larval**  **(%)** |
| --- | --- | --- | --- | --- | --- | --- |
| Ciudad Grande | | | | | | |
| Iquitos | 437,620 | 48 | 91.7 | 7.67 (9.46) | 0 | NA |
| Pucallpa | 310,750 | 140 | 52.9 | 1.83 (3.71) | 138 | 16.7 |
| Yurimaguas | 41,827 | 24 | 83.3 | 2.29 (2.05) | 24 | 33.3 |
| Ciudad Pequeña | | | | | | |
| Contamana | 17,429 | 76 | 75.0 | 5.21 (8.93) | 68 | 32.4 |
| Nauta | 19,551 | 67 | 88.1 | 5.16 (5.10) | 28 | 46.4 |
| Requena | 22,875 | 97 | 79.4 | 3.76 (6.14) | 28 | 28.6 |
| Tamshiyacu | 6,181 | 57 | 98.2 | 11.00 (12.84) | 30 | 63.3 |
| Pueblo | | | | | | |
| Bretaña * | 1,686 | 132 | 21.2 | 0.74 (2.66) | 109 | 14.7 |
| Flor de Punga | 1,763 | 78 | 79.2 | 3.77 (3.74) | 72 | 43.1 |
| Jenaro Herrera | 3,596 | 77 | 83.1 | 4.97 (8.20) | 26 | 42.3 |
| Juancito | 2,124 | 100 | 76.0 | 3.04 (4.46) | 62 | 41.9 |
| Maypuco | 1,248 | 24 | 83.3 | 2.58 (1.98) | 24 | 37.5 |
| Tamanco ☨ | 1,738 | 162 | 40.7 | 1.56 (3.26) | 154 | 29.9 |
| Tierra Blanca ☨ | 1,602 | 112 | 41.1 | 1.63 (3.51) | 109 | 36.7 |
| Caserío Ribereño | | | | | | |
| Aucayo | 587 | 65 | 84.6 | 8.48 (9.79) | 29 | 58.6 |
| Barrio Florida | 673 | 26 | 84.6 | 2.38 (2.80) | 26 | 46.2 |
| Canelos * | 317 | 60 | 23.3 | 0.72 (1.98) | 59 | 32.2 |
| Huacrachiro * | 667 | 87 | 62.1 | 3.15 (4.52) | 55 | 41.8 |
| La Pedrera | 531 | 65 | 38.5 | 3.62 (9.83) | 63 | 31.7 |
| Nuevo Paris / Nueva Alejandría | 361 | 45 | 0 | 0 | 45 | 0 |
| Santa Rosa de Masisea ☨ | 413 | 85 | 68.2 | 4.05 (7.48) | 83 | 34.9 |
| Tiruntan ☨ | 640 | 99 | 40.4 | 1.30 (3.12) | 99 | 32.3 |
| Tres Unidos | 507 | 62 | 24.2 | 1.39 (4.36) | 61 | 27.9 |
| Victoria * | 858 | 118 | 19.5 | 1.00 (3.30) | 29 | 20.7 |
| Caserío de Carretera | | | | | | |
| 1 de Febrero * | 183 | 13 | 30.8 | 1.23 (2.80) | 13 | 15.4 |
| 13 de Febrero * | 670 | 36 | 22.2 | 0.89 (2.05) | 24 | 12.5 |
| Cahuide * | 794 | 40 | 17.5 | 0.35 (0.92) | 40 | 5.0 |
| El Dorado * | 90 | 21 | 14.3 | 0.19 (0.51) | 21 | 4.8 |
| Nuevo Horizonte * | 308 | 21 | 9.5 | 0.14 (0.48) | 21 | 9.5 |
| San Jose * | 123 | 20 | 10.0 | 0.15 (0.49) | 20 | 10.0 |
| **^ζ^** Datos de ciudades grandes [38] y otros sitios [39, 40]  * primer reporte de presencia de *Ae. aegypti* en la comunidad | | | |  |  |  |
| ☨ primer brote de dengue ocurrió en la comunidad durante el año de las colectas | | | | |  |  |

*Distancia al puerto*

En los pueblos y caseríos ribereños donde muestreamos en transectos desde el puerto, muchas veces encontramos Ae. aegypti en las casas más cercanas a los puertos con más frecuencia que en las casas más alejadas del puerto (Fig. 5 y 6).

**Figura 5.** Los diagramas de violín muestran la distancia de cada hogar donde colectamos hacia el puerto comunitario y si Ae. aegypti estaba ausente o presente en la casa. Cada faceta muestra los resultados para un caserío o pueblo ribereño separado, limitado a aquellos sitios donde las colecciones se realizaron en transectos desde el puerto. La forma del diagrama de violín demuestra la densidad de hogares positivos y negativos para Ae. aegypti a lo largo de las distancias muestreadas. Se puede observar un patrón repetido: las casas sin Ae. aegypti tendían a concentrarse más lejos del puerto.

**Figura 6.** Mapas de cuatro sitios (3 caseríos ribereños y 1 pueblo – Flor de Punga). Los datos están superpuestos sobre imágenes satelitales de ESRI (2023 ESRI). Cada punto representa un evento de colecta en cada edificación. El color indica el número de Ae. aegypti colectados en la casa. Los puntos blancos representan casas donde el mosquito estaba ausente y los colores de azul a rojo indican un número de Ae. aegypti por casa cada vez mayor. En Tres Unidos, el caserío está dividido en dos barrios debido a desbarrancamiento (erosión resultando en secciones de la ribera cayendo al río), forzando a las personas a mudarse durante los últimos 5 años. El nuevo barrio estaba libre de Ae. aegypti, mientras que el barrio antiguo tenía Ae. aegypti concentrado cerca del puerto. De manera similar, en Victoria, Ae. aegypti estaba presente cerca del puerto y desaparecía en cierto punto dentro del pueblo. En contraste, Ae. aegypti estaba altamente disperso en Aucayo, aunque la mayoría de las casas negativas estaban ubicadas más lejos del puerto. En Flor de Punga, Ae. aegypti también estaba disperso por todo el pueblo, con las casas negativas dispersas sin un patrón claro. Mapas adicionales a nivel comunitario se pueden encontrar en la S1 Fig.

**Presencia de** Aedes aegypti
El patrón de distribución de mosquitos observado se investigó con un GLMM para determinar la relación entre la distancia al puerto y la probabilidad de presencia de Ae. aegypti (Fig. 7; Tabla C en S3 Tabla). En siete comunidades, las casas más cercanas al puerto tenían más probabilidades de ser positivas para Ae. aegypti que las casas más alejadas del puerto (p < 0.05). Cuatro comunidades tenían una relación marginal entre la distancia y la probabilidad de presencia de Ae. aegypti (0.05 ≤ p < 0.10). Tres comunidades no tenían relación entre la distancia al puerto y la probabilidad de presencia de Ae. aegypti.

**Figura 7.** El resultado del GLMM apoya una relación repetida entre la probabilidad predicha de presencia de Ae. aegypti en una casa y la distancia de la casa hacia el puerto comunitario. Cada línea representa la relación para cada una de las 14 comunidades incluidas en el análisis. El tipo de línea representa el nivel de significancia de la relación entre presencia y distancia en cada comunidad: la línea continua indica p < 0.05 (7 comunidades), la línea discontinua indica 0.05 ≤ p < 0.10 (4 comunidades), y la línea punteada indica p ≥ 0.10 (3 comunidades).

**Abundancia de** Aedes aegypti
Se utilizó un modelo glmmTMB con una distribución binomial negativa (elegida para tener en cuenta la sobredispersión de los datos) para determinar si existía una relación similar entre la distancia desde el puerto y el número de mosquitos adultos en la casa. No apareció ninguna tendencia clara (Tabla D en S3 Tabla): nueve de catorce comunidades no tenían una relación significativa entre la distancia y el número de Ae. aegypti, tres comunidades tenían una disminución significativa en el número de mosquitos más alejados del puerto y dos tenían un aumento significativo en los mosquitos en casas más alejadas del puerto.

**Discusión**
Nuestro estudio demuestra que Ae. aegypti ha invadido comunidades rurales a lo largo de una gran región de la Amazonía peruana, incluyendo numerosos caseríos remotos sin reportes previos de este vector. Nuestros resultados también sugieren un mecanismo de invasión: adultos de Ae. aegypti vuelen desde botes atracados en los puertos comunitarios. La extensión y ubicuidad de la invasión de Ae. aegypti es preocupante y representa una amenaza creciente para la salud pública por numerosas enfermedades transmitidas por vectores en la Amazonía rural.

Nuestras colectas muestran que la distancia desde una ciudad grande no limita la infestación de Ae. aegypti a lo largo de las principales rutas fluviales. Se encontró el vector en el sitio más lejano muestreado, Victoria, a 266 km de la ciudad más cercana, Iquitos. El único sitio donde Ae. aegypti estuvo ausente fue un par de comunidades cerca de Pucallpa. Esta dispersión generalizada, independiente de la distancia de una ciudad grande, difiere del patrón observado en 2011-2012, cuando se realizó el primer estudio sistemático de la expansión rural de Ae. aegypti en Perú en comunidades dentro de un radio de 95 km de Iquitos [21]. En ese momento, las comunidades con Ae. aegypti tendían a estar más cerca de Iquitos que las comunidades negativas para Ae. aegypti. Este cambio puede ser debido a la expansión del vector a áreas más remotas desde cuando se realizó el estudio original, modificando el patrón previamente observado. Alternativamente, la invasión a sitios más alejados de Iquitos podría haber ocurrido ya al momento del estudio de 2011-2012, pero, el patrón observado en ese estudio podría estar sesgado por los sitios seleccionados.

Nuestras colectas también muestran una expansión dramática de Ae. aegypti en los caseríos de carretera a lo largo de la carretera Iquitos-Nauta. Todos los seis caseríos muestreados estaban libres de Ae. aegypti durante las colecciones realizadas en 2011-2012. En 2023, todas las seis comunidades estaban infestadas con Ae. aegypti, abarcando todos 96 km de la carretera.

El nivel de infestación de Ae. aegypti en los caseríos rurales, pueblos y pequeñas ciudades fue sorprendente. Documentamos diez primeros reportes del mosquito en caseríos y pueblos rurales. Aunque hubo una tendencia de aumento en los niveles de infestación con el aumento de la urbanización, esta relación no fue perfectamente lineal y parece estar impulsada por los niveles de infestación particularmente bajos en los caseríos de carretera, así como por los niveles de infestación particularmente altos en las pequeñas ciudades. Hubo un notable nivel de variación en los niveles de infestación entre sitios dentro de la misma categoría de urbanización. También es notable que los índices de adultos de Ae. aegypti observadas en nuestro estudio superaron las reportadas en estudios previos realizados en Iquitos, Perú, con una media de Ae. aegypti colectados por casa rara vez superando 1 [41-44]. En algunos sitios rurales, el porcentaje de casas positivas para adultos de Ae. aegypti y el número promedio de Ae. aegypti por casa superaron los niveles de infestación en las grandes ciudades endémicas para el dengue, demostrando que los niveles de riesgo entomológico en algunas áreas rurales son suficientes para sostener un brote de dengue. Esto es particularmente sorprendente porque las ubicaciones de muestreo en las grandes ciudades fueron influenciadas por recomendaciones del departamento de salud sobre dónde había alta prevalencia de Ae. aegypti, lo que puede haber sesgado los resultados en las ciudades hacia niveles de infestación más altos de los que se encontrarían a través de muestreo aleatorio. El riesgo de transmisión de dengue en las comunidades rurales se ve aún más respaldado por el historial de brotes de dengue reportados en nueve de los pueblos y caseríos.

Dentro de los caseríos y pueblos rurales, observamos repetidamente que había mayor probabilidad encontrar los adultos de Aedes aegypti en casas más cercanas al puerto comunitario en comparación con casas más alejadas del puerto en siete de catorce comunidades, con una relación marginalmente significativa en cuatro comunidades adicionales. Este resultado apoya investigaciones previas en la región que atribuyen la dispersión a larga distancia a la infestación de Ae. aegypti en botes [23, 24]. También agrega un nivel de detalle: las invasiones probablemente ocurren en la etapa de vida adulta, no en las etapas de huevo o inmadura. La mayor probabilidad de presencia cerca de los puertos sugiere que las hembras adultas de Ae. aegypti vuelan desde los botes, oviponen en casas cercanas y las generaciones posteriores avanzan gradualmente adentro la comunidad, creando un patrón de distribución distinto que se irradia desde el puerto. Si la invasión ocurriera en las etapas de huevo o inmadura, esperaríamos una distribución de mosquitos más heterogénea, ya que los contenedores infestados deberían tener igual probabilidad de ser llevados a cualquier casa en la comunidad desde el barco. Aunque creemos que los patrones de distribución indican invasión en la etapa adulta, no es una prueba concluyente y puede tener otra explicación.

Hubo algunas excepciones a esta tendencia. En Juancito y Flor de Punga, que no tenían relación entre la distancia y la presencia de Ae. aegypti, la especie estaba ampliamente distribuida por todo el pueblo, con un AHI superior al 75%. El mosquito probablemente se estableció antes en estos sitios y completó su invasión en los pueblos antes de que muestreamos. Tres de las cuatro comunidades con una relación marginalmente significativa entre la presencia de Ae. aegypti y la distancia al puerto, también tenían una presencia de adultos con una distribución relativamente amplia (más del 60% AHI), lo que sugiere que la disminución del impacto de la distancia al puerto también puede deberse a que Ae. aegypti alcanzó el establecimiento. Esto se ve respaldado por evidencia previa de que el caserío de Aucayo ya estaba infestado con Ae. aegypti durante las colecciones de 2008 y 2012 [21]. Las únicas comunidades de bajo AHI sin una relación significativa entre la distancia y Ae. aegypti fueron Bretaña y Canelos. En Bretaña (AHI de 21.2%), había dos áreas focales de presencia, cerca del puerto y el centro del pueblo. Creemos que el foco cerca del centro del pueblo puede ser el único ejemplo de introducción en etapa de huevo o inmadura de Ae. aegypti entre nuestros sitios. Canelos (AHI 23.3%) tenía una relación marginal entre la distancia y la presencia de Ae. aegypti. El pueblo está ligeramente alejado del río y las casas con Ae. aegypti estaban en la mitad del pueblo más cercana al puerto, comenzando justo después de las casas más cercanas al puerto y terminando aproximadamente a mitad de camino a través del pueblo. No está claro si esta invasión ocurrió a través de etapas de vida adulta o de huevo/inmadura.

Dada la invasión global de Ae. aegypti, sorprendentemente hay poca información sobre la etapa de vida que causa nuevas invasiones. Las invasiones se han asociado con rutas de tránsito, pero mayormente no está claro si la invasión ocurrió a través de huevos, larvas o adultos importados [22, 45, 46]. En el caso del vector secundario del dengue, Ae. albopictus, hay mucha evidencia que los huevos y las larvas sirven como las etapas de vida invasoras, y en menor medida, los adultos a través del transporte pasivo en vehículos terrestres [47-49]. La evidencia de dispersión pasiva de mosquitos adultos a través de botes presentada por este estudio es única en su nivel de detalle y replicación en múltiples sitios.

Los patrones de distribución a nivel comunitario también muestran que Ae. aegypti se encuentra en diferentes etapas de invasión en distintas comunidades. En algunas ubicaciones está bien establecido, mientras que, en otras, parece estar comenzando su invasión cerca del puerto. Alternativamente, este patrón de distribución puede sugerir una invasión fallida, en la cual el mosquito no ha podido avanzar más adentro de la comunidad. Sin embargo, esto parece poco probable porque, anecdóticamente, los pueblos no tienen barreras internas de expansión. También es posible que las diferencias entre comunidades hayan sido un artefacto de la fecha de colecta y de los cambios estacionales en el clima, ya que cada comunidad fue muestreada solo una vez durante el período de seis meses. Sin embargo, no creemos que este sea el caso, dado que las colectas se realizaron durante la temporada de lluvias y se detectaron tanto niveles bajos como altos de infestación a lo largo del período de muestreo, sin estar agrupados por fecha. La identificación de estas invasiones activas proporciona una rara oportunidad para monitorear y entender el proceso de invasión en tiempo real.

La distribución amplia de Ae. aegypti en la Amazonía rural tiene importantes implicaciones para la salud pública. Más de un millón de personas viven en comunidades rurales en la Amazonía peruana [39, 40, 50-52]. Con la llegada de Ae. aegypti, estas comunidades rurales están ahora en riesgo contagiarse con los virus del dengue, Zika y chikungunya. Muchas de las comunidades están ubicadas lejos de hospitales [25, 26], con algunos sitios del estudio a más de 18 horas en bote rápido, que viaja intermitentemente. Las comunidades a lo largo de ríos menos transitados enfrentan desafíos de acceso aún mayores. Como resultado de esta inaccesibilidad, los casos severos de dengue que requieren atención hospitalaria inmediata son evacuados en avión, un esfuerzo costoso que consume tiempo. La detección temprana, la hospitalización oportuna y la atención adecuada son críticas para la supervivencia de los pacientes hemorrágicos, todos los cuales son limitados en áreas rurales [53]. La creciente amenaza del dengue en las comunidades rurales podría resultar en una carga desproporcionada de enfermedad severa en comparación con las áreas urbanas debido a la inaccesibilidad de la atención.

La invasión amplia de Ae. aegypti a la Amazonía rural también aumenta el riesgo de que el virus de la fiebre amarilla silvestre (YFV) entre en un ciclo de transmisión urbana. Un gran brote de fiebre amarilla ocurrió en varias regiones de Perú en 1995 [54, 55], y entre 2000 y 2014, Perú representó el 37.4% de los casos reportados de fiebre amarilla en las Américas [56]. Se supone que la deforestación aumenta los brotes de fiebre amarilla debido a la bajada de los vectores Haemogogus al suelo, los cuales normalmente habitan las copas de los árboles, aumentando el contacto mosquito-humano [55]. Esta hipótesis se investigó en un estudio ecológico, que no logró descubrir una relación entre la actividad humana (por ejemplo, la pérdida de árboles) y la incidencia de casos de fiebre amarilla, pero los datos pueden haber sido demasiado imprecisos para capturar la relación con precisión [56]. Los cambios recientes en la ley peruana permiten más deforestación en la Amazonía [57], lo que podría aumentar la transmisión de fiebre amarilla a personas en caseríos remotos, si la deforestación realmente aumenta la exposición humana. La presencia de Ae. aegypti en áreas rurales aumenta el riesgo de amplificación del YFV en poblaciones humanas rurales, aumentando así también la probabilidad de transmisión urbana al aumentar el número de posibles conductos hacia la ciudad. Esto cambia la comprensión actual del ciclo de transmisión rural del YFV como dependiente de mosquitos generalistas [58], y puede expandir significativamente el riesgo de amplificación. También es probable que se produzca un aumento similar del riesgo para otros arbovirus emergentes, como el virus Mayaro [59].

Nuestro estudio muestra un nivel preocupante de expansión de Ae. aegypti hacia la Amazonía rural y destaca la necesidad de prestar atención a esta nueva amenaza para la salud rural. Dado el riesgo para la salud pública demostrado por nuestro data, alertamos de inmediato a las autoridades sanitarias regionales, lo que ha llevado a los esfuerzos del departamento de salud para expandir la vigilancia de Ae. aegypti fuera de las ciudades. Sin embargo, esta vigilancia ampliada aún se concentrará en pueblos más grandes debido a recursos limitados.

Todavía hay mucho por aprender sobre este proceso de invasión. Para nuestro estudio, seleccionamos sitios a lo largo de la principal ruta fluvial; la mayoría son relativamente grandes en comparación con otros caseríos de la región, con un mayor grado de conexión de tránsito fluvial con las ciudades. Estas características pueden hacer que nuestros sitios sean más propensos a ser invadidos por Ae. aegypti que comunidades más pequeñas y menos conectadas. Los estudios futuros deberían centrarse en comunidades más pequeñas y menos conectadas para determinar si ciertas características a nivel comunitario influyen en el establecimiento de Ae. aegypti. También debería investigar la transmisión del dengue en comunidades rurales. Y finalmente, hay una necesidad urgente de identificar estrategias para controlar las poblaciones de *Ae. aegypti* en zonas rurales dentro de la región y detener la expansión adicional de la especie. Nuestra investigación sugiere que todavía hay tiempo para intervenir antes de que el mosquito se establezca completamente en muchas comunidades rurales de la región.

**Agradecimientos:** Gracias a todos los que hicieron posible esta investigación, especialmente a las comunidades que nos recibieron en sus hogares y nos permitieron colectar en sus casas. Gracias a la Dra. Erika Mudrak (Cornell Statistical Consulting Unit) por su asesoramiento en el análisis estadístico, a Pilar Díaz (GERESA Loreto) por proporcionar datos sobre el uso histórico de insecticidas por GERESA en las comunidades, y a Gabriela Vásquez La Torre (Prisma) y al Dr. Helvio Astete (NAMRU SOUTH) por el apoyo logístico y el asesoramiento. Nuestro agradecimiento sincero al Centro Atkinson para la Sostenibilidad de Cornell por el apoyo financiero a través de los Rapid Response Fund y Academic Venture Fund.

**Descargo de responsabilidad**
Las opiniones expresadas en este artículo reflejan los resultados de la investigación realizada por los autores y no representan necesariamente la política o posición oficial del Department of the Navy, Department of Defense ni del Gobierno de los Estados Unidos.

**Declaración de derechos de autor**
Dos de los autores son empleados del gobierno de los Estados Unidos. Este trabajo fue preparado como parte de sus funciones oficiales. El Título 17 del Código de los Estados Unidos (U.S.C.) §105 establece que la protección de derechos de autor bajo este título no está disponible para ningún trabajo del Gobierno de los Estados Unidos. El Título 17 U.S.C. §101 define una obra del Gobierno de los EE. UU. como una obra preparada por un miembro del servicio militar o un empleado del Gobierno de los EE. UU. como parte de sus funciones oficiales.

**Información Suplementaria:**

Tabla suplementaria S1. Características del sitio. *Características de cada sitio, incluyendo departamento, población, tamaño del sitio, historial de brotes de dengue y fase de recolección.*

Documento Suplementario S2. Involucramiento Comunitario. *Este documento presenta los métodos utilizados para realizar actividades de alcance comunitario e involucrar a los miembros de las comunidades en el proceso científico.*

Documento Suplementario S3. Métodos y Resultados de la Encuesta de Hábitats Larvales. *Este documento describe los métodos utilizados para recolectar y analizar datos sobre hábitats larvales, así como los resultados y discusión de los resultados.*

Tabla Suplementaria S4. Detalles de los Modelos Mixtos Lineales Generalizados (GLMM). *Esta tabla detalla los cuatro GLMM utilizados en este estudio, incluyendo la variable de respuesta, familia, efectos fijos y aleatorios, número de sitios, número de observaciones y otras notas.*

Documento Suplementario S5. Traducción al Español // Spanish Translation. *Este documento proporciona una traducción del artículo al español para mejorar la accesibilidad // This document provides a translation of the paper to Spanish to improve accessibility.*

Tabla Suplementaria S6. Resultados de Emmeans y Emtrends para los GLMM. *Cuatro tablas que reportan los resultados de las comparaciones por pares y las tendencias para los cuatro modelos realizados en este estudio.*

Figura Suplementaria S7. Mapas de Sitios y Datos de Colecta. *Imágenes satelitales de los 30 sitios, con puntos de datos superpuestos que representan el número de Ae. aegypti adultos capturados durante cada evento de colecta en viviendas.*

**Referencias**

1. Paz-Bailey G, Adams LE, Deen J, Anderson KB, Katzelnick LC. Dengue. The Lancet. 2024;403(10427):667-82.

2. Bhatt S, Gething PW, Brady OJ, Messina JP, Farlow AW, Moyes CL, et al. The global distribution and burden of dengue. Nature. 2013;496(7446):504.

3. Shepard DS, Undurraga EA, Halasa YA, Stanaway JD. The global economic burden of dengue: a systematic analysis. The Lancet infectious diseases. 2016;16(8):935-41.

4. Soper FL. The elimination of urban yellow fever in the Americas through the eradication of Aedes aegypti. American Journal of Public Health and the Nations Health. 1963;53(1):7-16.

5. Phillips I, Need J, Escamilla J, Colán E, Sánchez S, Rodríguez M, et al. First documented outbreak of dengue in the Peruvian Amazon region. Bulletin of the Pan American Health Organization (PAHO); 26 (3), 1992. 1992.

6. San Martín J, Montoya RH, del Diego J, Zambrano B, Dayan GH. The history of dengue outbreaks in the Americas. The American journal of tropical medicine and hygiene. 2012;87(4):584-93.

7. Kolimenakis A, Heinz S, Wilson ML, Winkler V, Yakob L, Michaelakis A, et al. The role of urbanisation in the spread of Aedes mosquitoes and the diseases they transmit—A systematic review. PLoS Neglected Tropical Diseases. 2021;15(9):e0009631.

8. Man O, Kraay A, Thomas R, Trostle J, Lee GO, Robbins C, et al. Characterizing dengue transmission in rural areas: A systematic review. PLoS Neglected Tropical Diseases. 2023;17(6):e0011333.

9. Olano VA. *Aedes aegypti* en el área rural: implicaciones en salud pública. Biomédica. 2016;36(2):169-73.

10. Overgaard HJ, Olano VA, Jaramillo JF, Matiz MI, Sarmiento D, Stenström TA, et al. A cross-sectional survey of *Aedes aegypti* immature abundance in urban and rural household containers in central Colombia. Parasites & vectors. 2017;10:1-12.

11. Marquetti MdC, Bisset J, Leyva M, García A, Rodríguez M. Comportamiento estacional y temporal de *Aedes aegypti* y *Aedes albopictus* en La Habana, Cuba. Revista Cubana de Medicina Tropical. 2008;60(1):0-.

12. Rubio-Palis Y, Guzmán H, Espinoza J, Cárdenas L, Bevilacqua M, Medina D. Primer registro de *Aedes* (Stegomyia) *aegypti* (L.) en áreas remotas del estado Bolívar. Boletín De Malariología Y Salud Ambiental. 2011;51(1):89-91.

13. Troyes L, Villegas Z, Troyes M. Expansión del *Aedes aegypti* a localidades rurales de Cajamarca. Revista Peruana de Medicina Experimental y Salud Publica. 2006;23(3):163-7.

14. Pérez-Castro R, Castellanos JE, Olano VA, Matiz MI, Jaramillo JF, Vargas SL, et al. Detection of all four dengue serotypes in *Aedes aegypti* female mosquitoes collected in a rural area in Colombia. Memorias do Instituto Oswaldo Cruz. 2016;111:233-40.

15. Velandia-Romero ML, Olano VA, Coronel-Ruiz C, Cabezas L, Calderón-Peláez MA, Castellanos JE, et al. Detección del virus del dengue en larvas y pupas de *Aedes aegypti* recolectadas en áreas rurales del municipio de Anapoima, Cundinamarca, Colombia. Biomédica. 2017;37(2):193-200.

16. Lee GO, Vasco L, Márquez S, Zuniga-Moya JC, Van Engen A, Uruchima J, et al. A dengue outbreak in a rural community in Northern Coastal Ecuador: An analysis using unmanned aerial vehicle mapping. PLoS neglected tropical diseases. 2021;15(9):e0009679.

17. Márquez S, Carrera J, Espín E, Cifuentes S, Trueba G, Coloma J, et al. Dengue Serotype Differences in Urban and Semi-rural Communities in Ecuador. ACI Avances en Ciencias e Ingenierías. 2018;10(1).

18. Charette M, Berrang-Ford L, Llanos-Cuentas EA, Cárcamo C, Kulkarni M. What caused the 2012 dengue outbreak in Pucallpa, Peru? A socio-ecological autopsy. Social Science & Medicine. 2017;174:122-32.

19. Fernández W, Iannacone J, Rodríguez E, Salazar N, Valderrama B, Morales AM. Comportamiento poblacional de larvas de aedes egypti para estimar los casos de dengue en Yurimaguas, Perú, 2000-2004. Revista peruana de medicina experimental y Salud Pública. 2005;22(3):175-82.

20. Morrison AC, Minnick SL, Rocha C, Forshey BM, Stoddard ST, Getis A, et al. Epidemiology of dengue virus in Iquitos, Peru 1999 to 2005: interepidemic and epidemic patterns of transmission. PLoS neglected tropical diseases. 2010;4(5):e670.

21. Guagliardo SA, Barboza JL, Morrison AC, Astete H, Vazquez-Prokopec G, Kitron U. Patterns of geographic expansion of *Aedes aegypti* in the Peruvian Amazon. PLoS neglected tropical diseases. 2014;8(8):e3033.

22. Guagliardo SAJ, Lee Y, Pierce AA, Wong J, Chu YY, Morrison AC, et al. The genetic structure of *Aedes aegypti* populations is driven by boat traffic in the Peruvian Amazon. Plos Neglect Trop Dis. 2019;13(9):e0007552.

23. Guagliardo SA, Morrison AC, Barboza JL, Requena E, Astete H, Vazquez-Prokopec G, et al. River Boats Contribute to the Regional Spread of the Dengue Vector *Aedes aegypti* in the Peruvian Amazon. Plos Neglect Trop Dis. 2015;9(4). doi: 10.1371/journal.pntd.0003648. PubMed PMID: WOS:000354972200022.

24. Guagliardo SA, Morrison AC, Barboza JL, Wesson DM, Ponnusamy L, Astete H, et al. Evidence for *Aedes aegypti* (Diptera: Culicidae) Oviposition on Boats in the Peruvian Amazon. J Med Entomol. 2015;52(4):726-9. doi: 10.1093/jme/tjv048. PubMed PMID: WOS:000357688400026.

25. Limaye NP, Blas MM, Alva IE, Carcamo CP, García PJ. The Amazon Hope: A qualitative and quantitative assessment of a mobile clinic ship in the Peruvian Amazon. PloS one. 2018;13(6):e0196988.

26. Brierley CK, Suarez N, Arora G, Graham D. Healthcare access and health beliefs of the indigenous peoples in remote Amazonian Peru. The American journal of tropical medicine and hygiene. 2014;90(1):180.

27. Nacional IG. Límites Departamentales. 2023.

28. (ROLAC) OLAatC. Hidrografía de Perú. 2015.

29. Vazquez-Prokopec GM, Galvin WA, Kelly R, Kitron U. A new, cost-effective, battery-powered aspirator for adult mosquito collections. J Med Entomol. 2009;46(6):1256-9.

30. Darsie RF, Ward RA. Identification and geographical distribution of the mosquitoes of North America, north of Mexico: University Press of Florida Gainesville, FL; 2005.

31. Loreto Censos Nacionales de Población y Vivienda 2017. Instituto Nacional de Estadística e Informática. 2017.

32. Ucayali Censos Nacionales de Población y Vivienda 2017. Instituto Nacional de Estadística e Informática. 2017.

33. RCoreTeam. R: A Language and Environment for Statistical Computing. 2019.

34. Lenth R. emmeans: estimated marginal means, aka least-squares means. R package v. 1.3. 4. 2019.

35. Bates D, Mächler M, Bolker B, Walker S. Fitting linear mixed-effects models using lme4. arXiv preprint arXiv:14065823. 2014.

36. Magnusson A, Skaug H, Nielsen A, Berg C, Kristensen K, Maechler M, et al. Package ‘glmmtmb’. R Package Version 02 0. 2017.

37. Fikrig K, Arnold O. Noriega, Rosa A. Rodriguez, John Bardales, José Rivas, Becker Reyna, Guido Izquierdo, Gissella M. Vasquez, Ryan T. Larson, Amy Morrison, and Laura Harrington. . Data from: Changing dynamics of Aedes aegypti invasion and vector-borne disease risk for rural communities in the Peruvian Amazon. [dataset]. Cornell University eCommons Repository. 2025.

38. Review WP. Population of Cities in Peru 2024. 2024.

39. Ucayali Resultados Definitivos. Instituto Nacional de Estadística e Informática. 2018.

40. Loreto Resultados Definitivos. Instituto Nacional de Estadística e Informática. 2018.

41. Morrison A, Astete H, Chapilliquen F, Ramirez-Prada G, Diaz G, Getis A, et al. Evaluation of a sampling methodology for rapid assessment of *Aedes aegypti* infestation levels in Iquitos, Peru. Journal of medical entomology. 2004;41(3):502-10.

42. Cromwell EA, Stoddard ST, Barker CM, Van Rie A, Messer WB, Meshnick SR, et al. The relationship between entomological indicators of *Aedes aegypti* abundance and dengue virus infection. PLoS neglected tropical diseases. 2017;11(3):e0005429.

43. Gunning CE, Okamoto KW, Astete H, Vasquez GM, Erhardt E, Del Aguila C, et al. Efficacy of *Aedes aegypti* control by indoor Ultra Low Volume (ULV) insecticide spraying in Iquitos, Peru. PLoS neglected tropical diseases. 2018;12(4):e0006378.

44. Morrison AC, Reiner Jr RC, Elson WH, Astete H, Guevara C, Del Aguila C, et al. Efficacy of a spatial repellent for control of *Aedes*-borne virus transmission: A cluster-randomized trial in Iquitos, Peru. Proceedings of the National Academy of Sciences. 2022;119(26):e2118283119.

45. Fonzi E, Higa Y, Bertuso AG, Futami K, Minakawa N. Human-mediated marine dispersal influences the population structure of *Aedes aegypti* in the Philippine Archipelago. PLoS neglected tropical diseases. 2015;9(6):e0003829.

46. Diaz-Nieto LM, Chiappero MB, de Astarloa CD, Macia A, Gardenal CN, Beron CM. Genetic Evidence of Expansion by Passive Transport of *Aedes* (Stegomyia) *aegypti* in Eastern Argentina. PLoS Neglected Tropical Diseases. 2016;10(9). doi: 10.1371/journal.pntd.0004839. PubMed PMID: WOS:000385627900003.

47. Hawley WA, Reiter P, Copeland RS, Pumpuni CB, Craig Jr GB. *Aedes albopictus* in North America: probable introduction in used tires from northern Asia. Science. 1987;236(4805):1114-6.

48. Reiter P, Sprenger D. The used tire trade: a mechanism for the worldwide dispersal of container breeding mosquitoes. 1987.

49. Ibáñez-Justicia A. Pathways for introduction and dispersal of invasive *Aedes* mosquito species in Europe: a review. Journal of the European Mosquito Control Association. 2020;38(1-10).

50. Madre de Dios Resultados Definitivos. Instituto Nacional de Estadística e Informática. 2018.

51. San Martín Resultados Definitivos. Instituto Nacional de Estadística e Informática. 2018.

52. Huánuco Resultados Definitivos. Instituto Nacional de Estadística e Informática. 2018.

53. Guha-Sapir D, Schimmer B. Dengue fever: new paradigms for a changing epidemiology. Emerging themes in epidemiology. 2005;2(1):1-10.

54. Robertson SE, Hull BP, Tomori O, Bele O, LeDuc JW, Esteves K. Yellow fever: a decade of reemergence. Jama. 1996;276(14):1157-62.

55. Bryant J, Wang H, Cabezas C, Ramirez G, Watts D, Russell K, et al. Enzootic transmission of yellow fever virus in Peru. Emerging infectious diseases. 2003;9(8):926.

56. Hamrick PN, Aldighieri S, Machado G, Leonel DG, Vilca LM, Uriona S, et al. Geographic patterns and environmental factors associated with human yellow fever presence in the Americas. PLoS neglected tropical diseases. 2017;11(9):e0005897.

57. Martel C, Mendieta-Leiva G, Alvarez-Loayza PC, Cano A, Cosio EG, Decock C, et al. Peru’s zoning amendment endangers forests. Science. 2024;383(6686):957-.

58. Gabiane G, Yen PS, Failloux AB. *Aedes* mosquitoes in the emerging threat of urban yellow fever transmission. Reviews in Medical Virology. 2022;32(4):e2333.

59. Mackay IM, Arden KE. Mayaro virus: a forest virus primed for a trip to the city? Microbes and infection. 2016;18(12):724-34.
