# Supplementary material for: Changing dynamics of Aedes aegypti invasion and vector-borne disease risk for rural communities in the Peruvian Amazon
Source: PLoS Negl Trop Dis. 2025 Aug 28;19(8):e0012506. doi: 10.1371/journal.pntd.0012506 (PMC12393723; doi:10.1371/journal.pntd.0012506)

S1 FIG.
MAPS OF SITES AND COLLECTION DATA

This document contains 30 maps of each of the sites in the associated publication, “Changing dynamics of *Aedes aegypti* invasion and vector-borne disease risk for rural communities in the Peruvian Amazon”. Data is overlaid on ESRI satellite imagery (2023 ESRI) using qGIS version 3.40.4. Each point represents a household collection event. The color indicates the number of *Ae. aegypti* collected in the house. White points represent houses where the mosquito was absent and colors from blue to red indicate an increasing number of *Ae. aegypti* per house. The sites are grouped by river or road (Amazon, Ucayali, Puinahua, and Marañon/Huallaga Rivers, and Iquitos-Nauta road).

MAPS OF SITES ON THE AMAZON RIVER

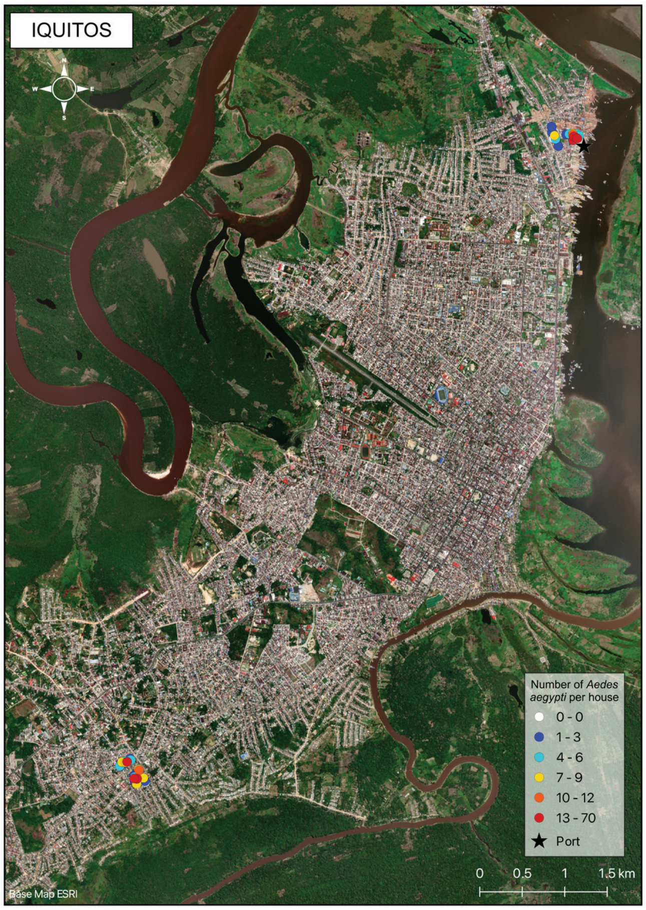

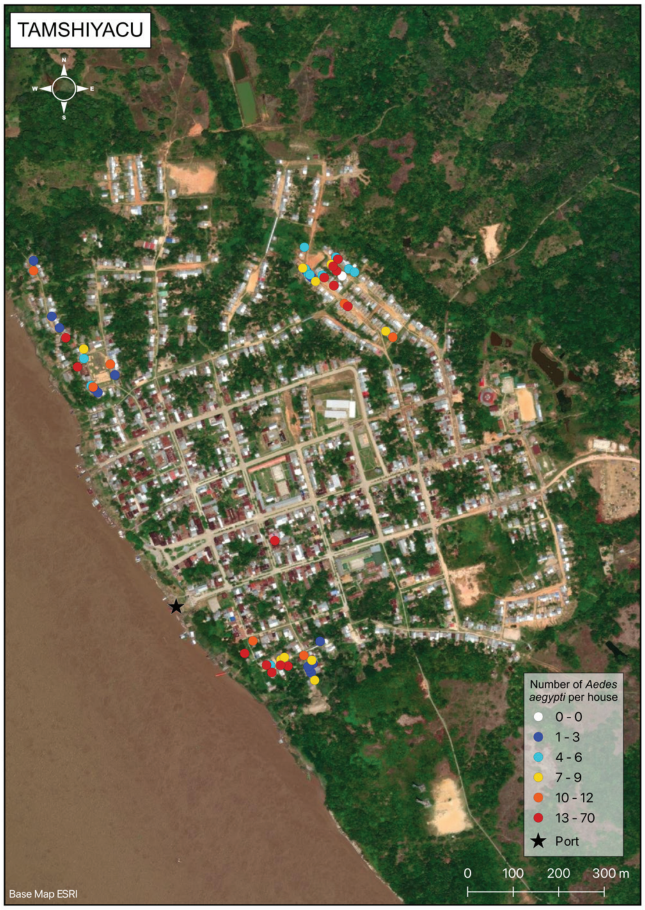

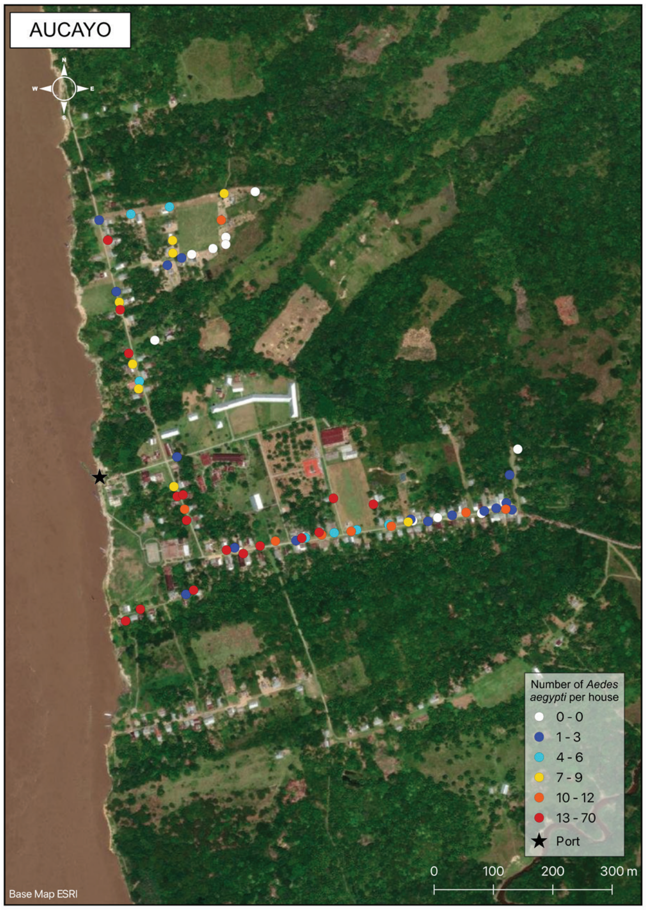

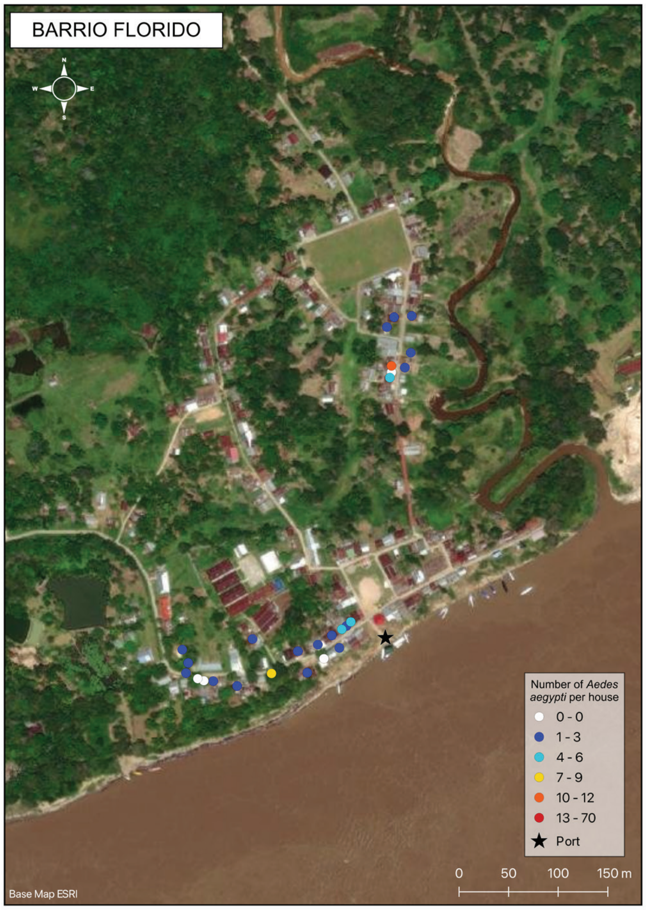

MAPS OF SITES ON THE UCAYALI RIVER

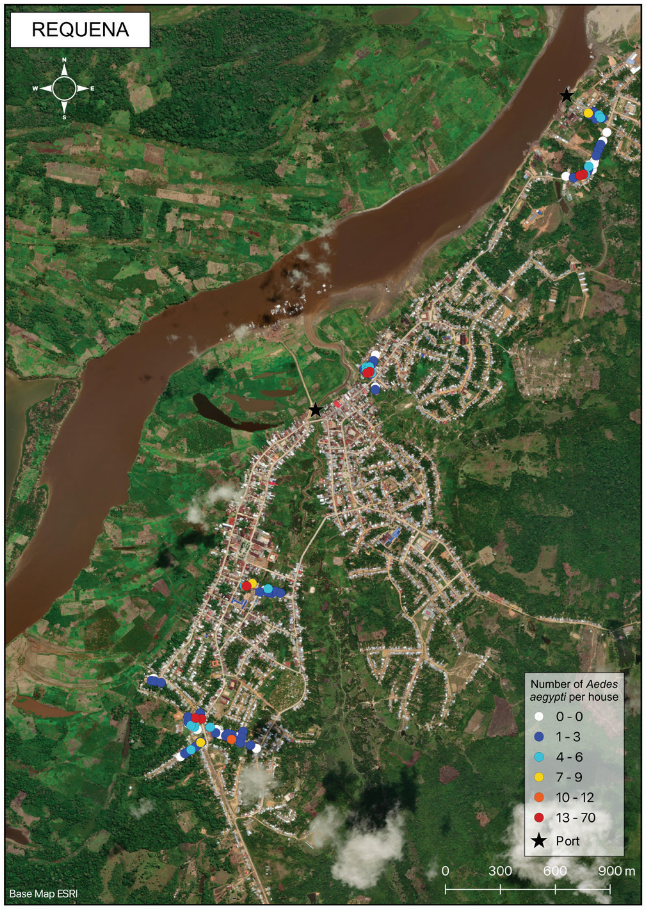

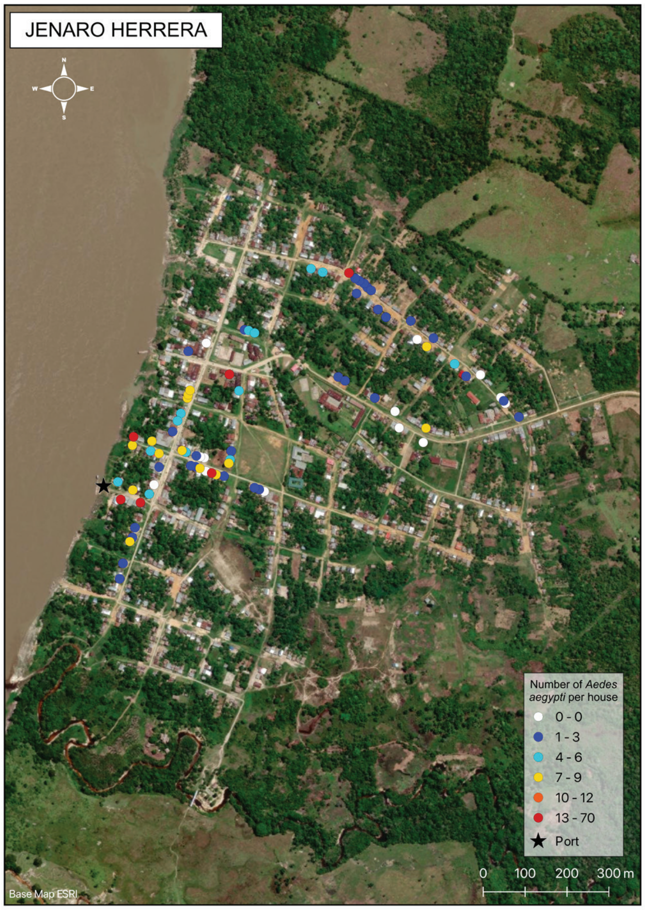

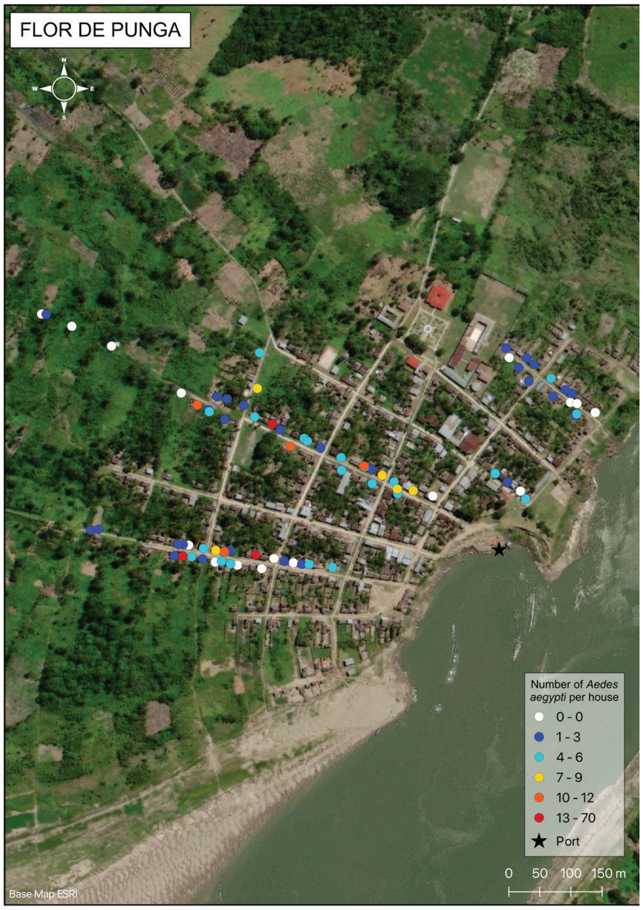

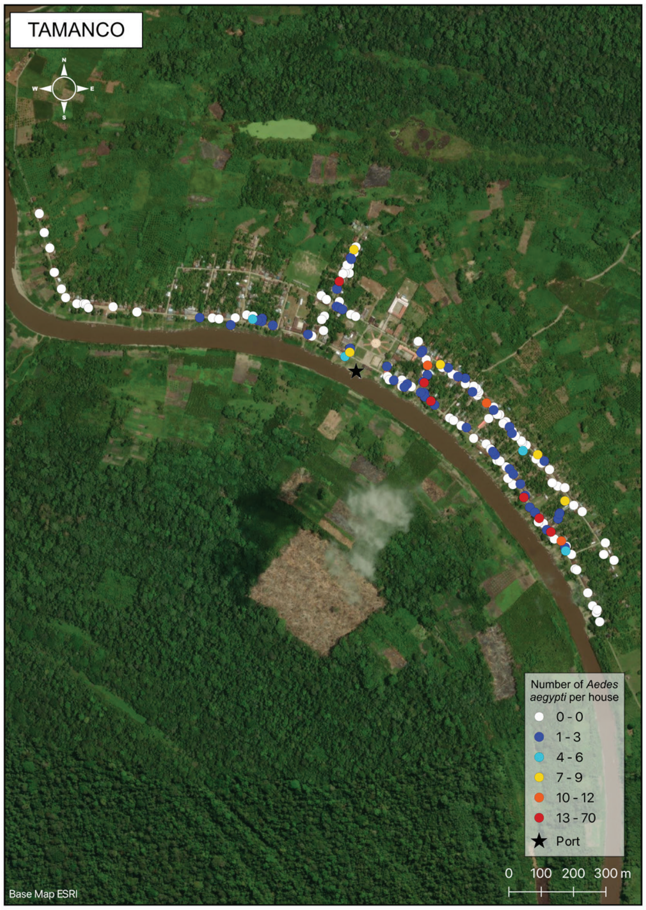

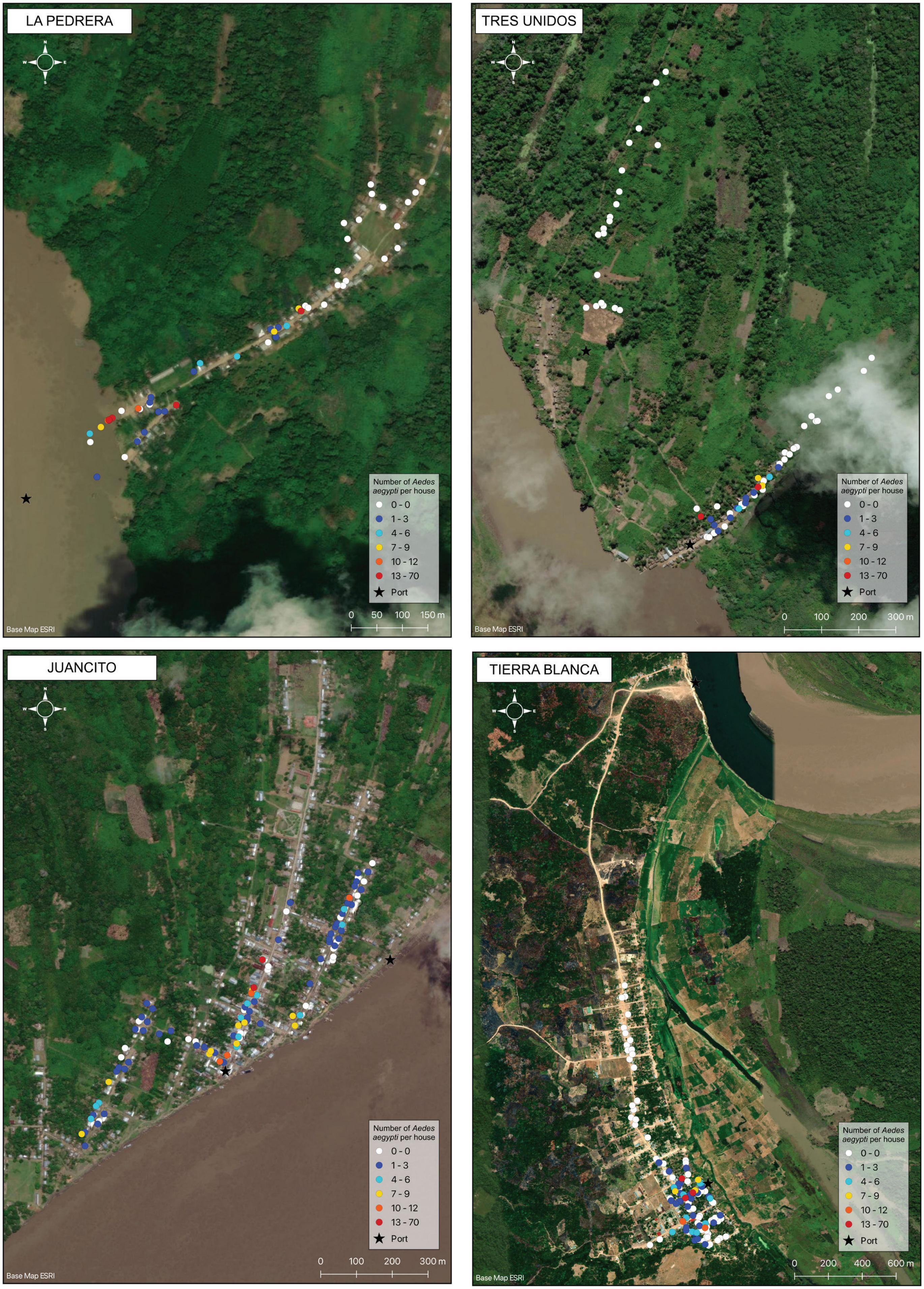
LA PEDRERA
Number of Aedes aegypti per house
0 - 0
1 - 3
4 - 6
7 - 9
10 - 12
13 - 70
Port
0 50 100 150 m
Base Map ESRI
TRES UNIDOS
Number of Aedes aegypti per house
0 - 0
1 - 3
4 - 6
7 - 9
10 - 12
13 - 70
Port
0 100 200 300 m
Base Map ESRI
JUANCITO
Number of Aedes aegypti per house
0 - 0
1 - 3
4 - 6
7 - 9
10 - 12
13 - 70
Port
0 100 200 300 m
Base Map ESRI
TIERRA BLANCA
Number of Aedes aegypti per house
0 - 0
1 - 3
4 - 6
7 - 9
10 - 12
13 - 70
Port
0 200 400 600 m
Base Map ESRI

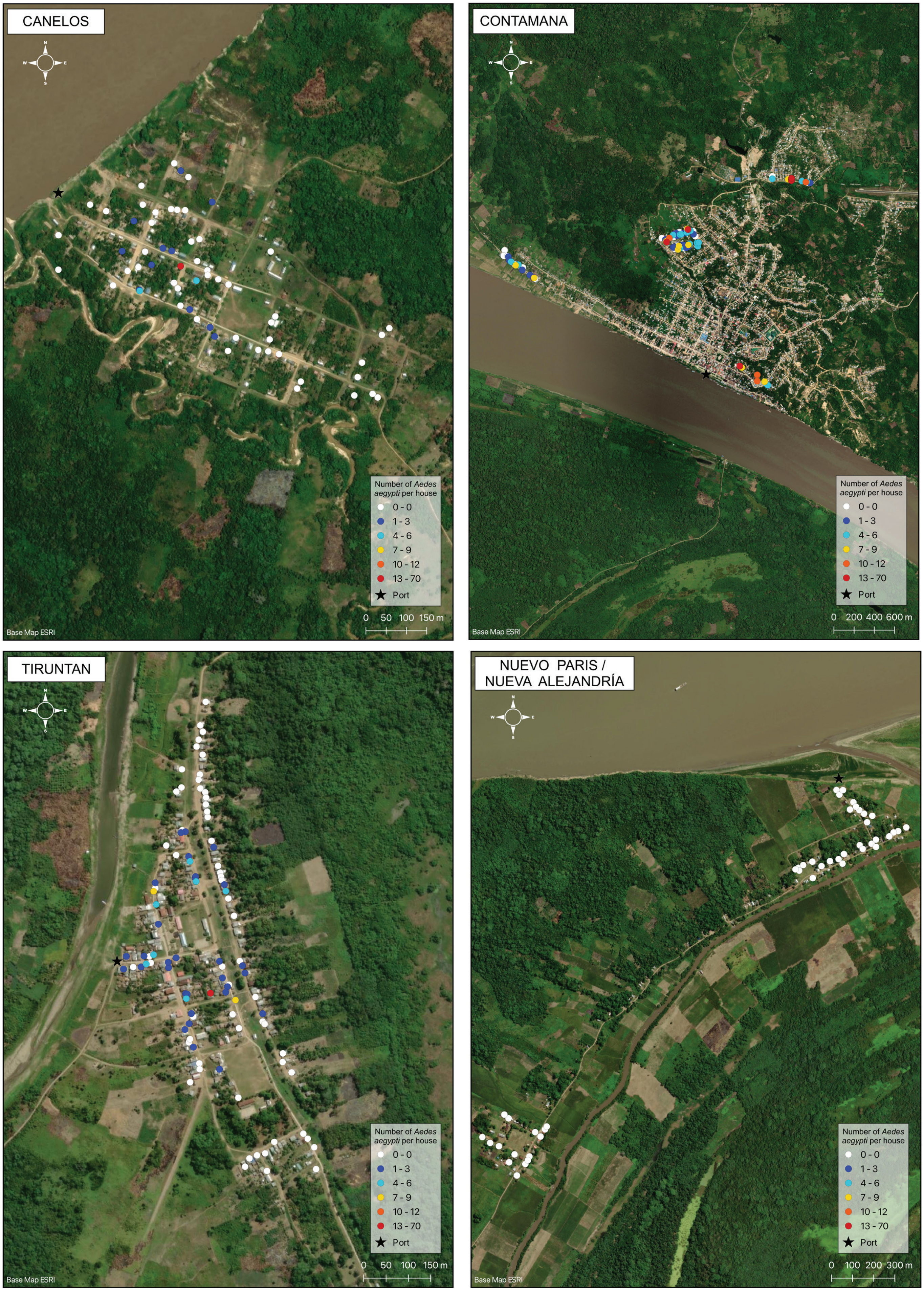
CANELOS
Number of *Aedes aegypti* per house
0 - 0
1 - 3
4 - 6
7 - 9
10 - 12
13 - 70
Port
0 50 100 150 m
Base Map ESRI
CONTAMANA
Number of *Aedes aegypti* per house
0 - 0
1 - 3
4 - 6
7 - 9
10 - 12
13 - 70
Port
0 200 400 600 m
Base Map ESRI
TIRUNTAN
Number of *Aedes aegypti* per house
0 - 0
1 - 3
4 - 6
7 - 9
10 - 12
13 - 70
Port
0 50 100 150 m
Base Map ESRI
NUEVO PARIS /
NUEVA ALEJANDRÍA
Number of *Aedes aegypti* per house
0 - 0
1 - 3
4 - 6
7 - 9
10 - 12
13 - 70
Port
0 100 200 300 m
Base Map ESRI

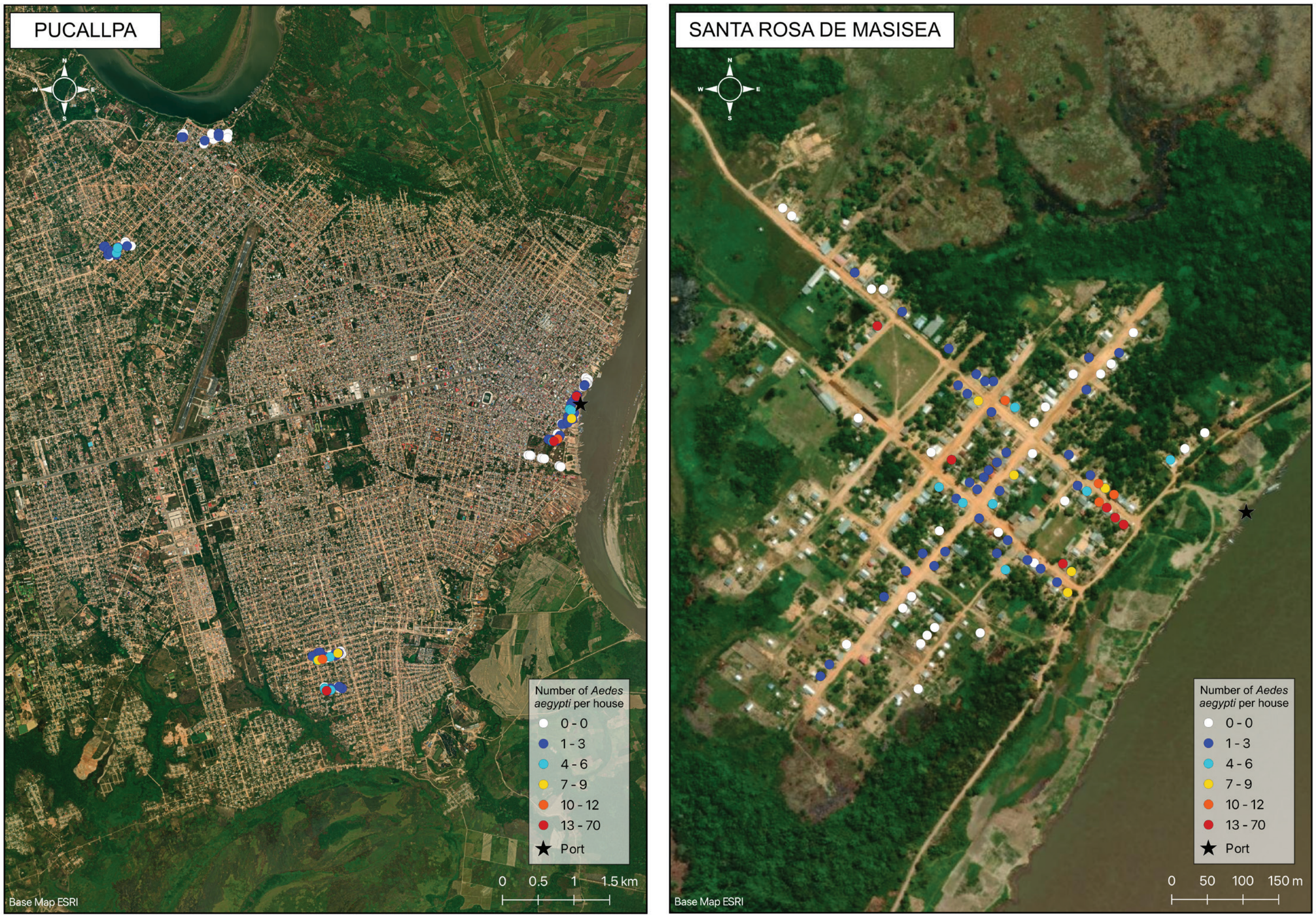

PUCALLPA
Number of Aedes aegypti per house
0 - 0
1 - 3
4 - 6
7 - 9
10 - 12
13 - 70
Port
0 0.5 1 1.5 km
Base Map ESRI
SANTA ROSA DE MASISEA
Number of Aedes aegypti per house
0 - 0
1 - 3
4 - 6
7 - 9
10 - 12
13 - 70
Port
0 50 100 150 m
Base Map ESRI

MAPS OF SITES ON THE PUINAHUA RIVER

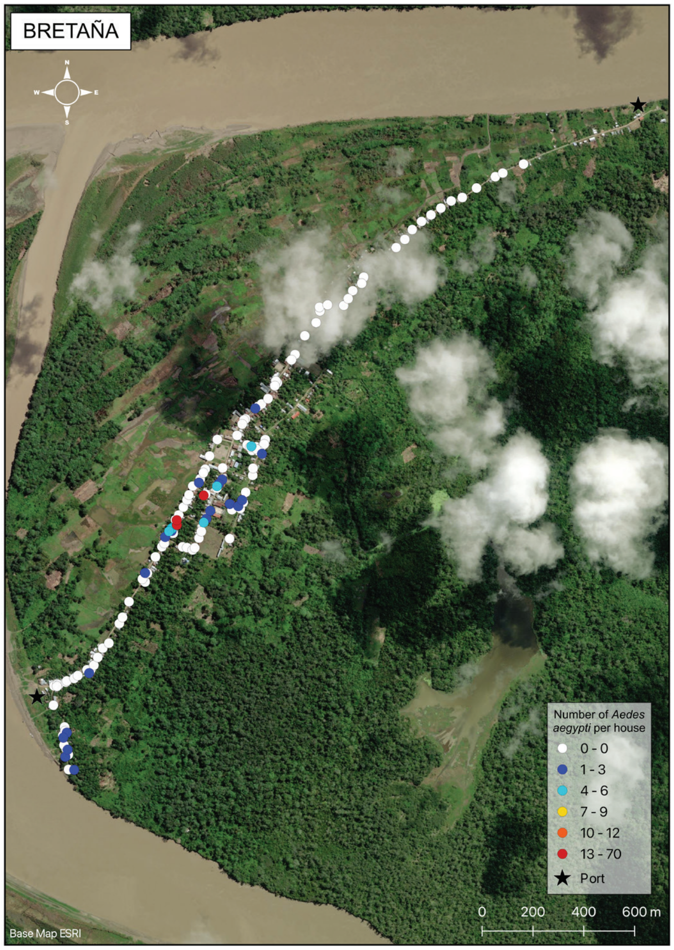

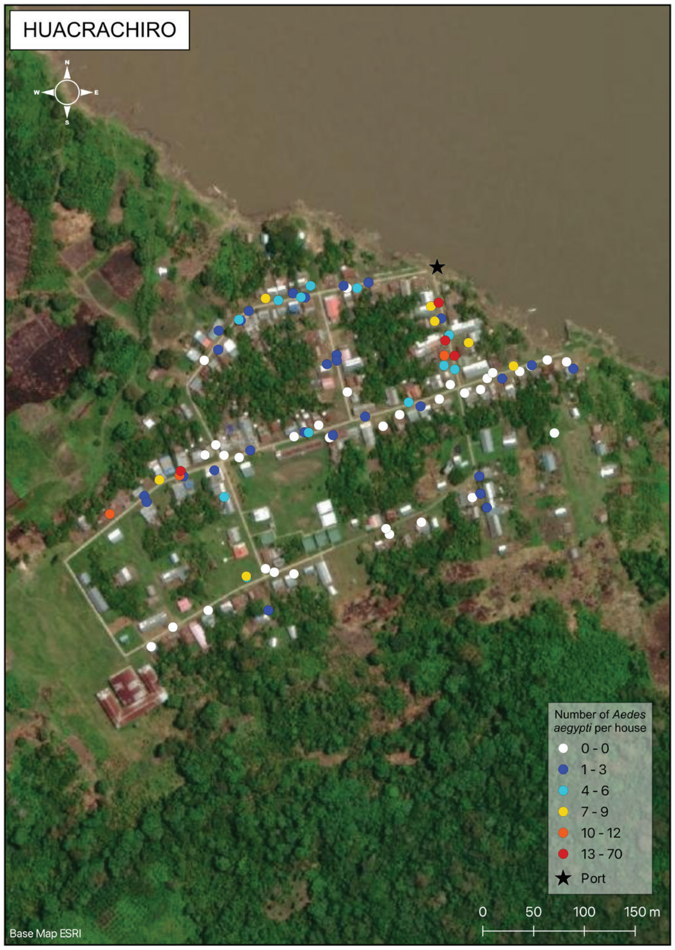

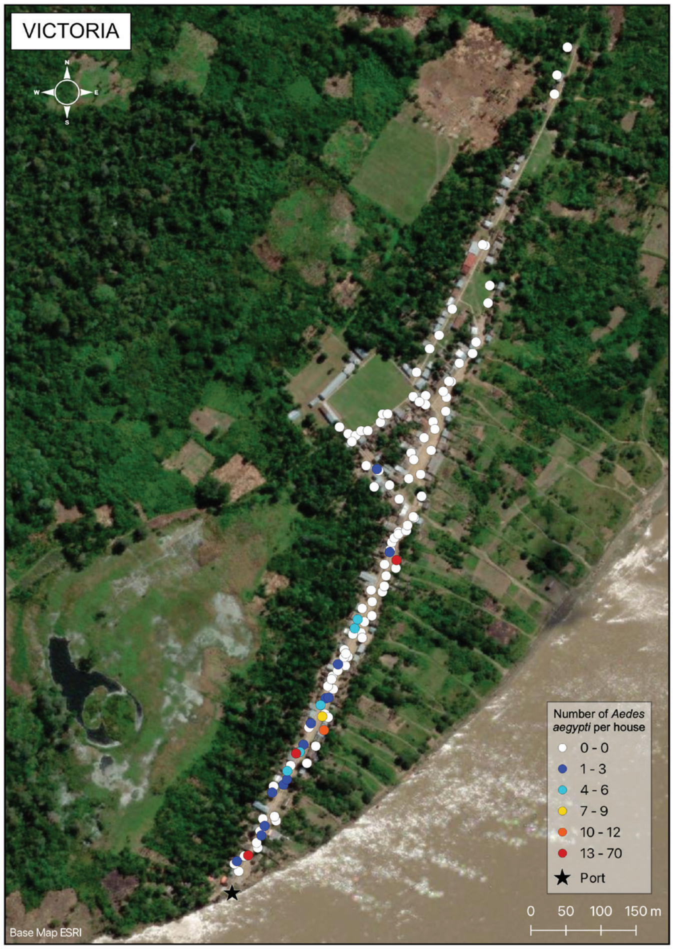

MAPS OF SITES ON THE MARAÑON AND HUALLAGA RIVERS

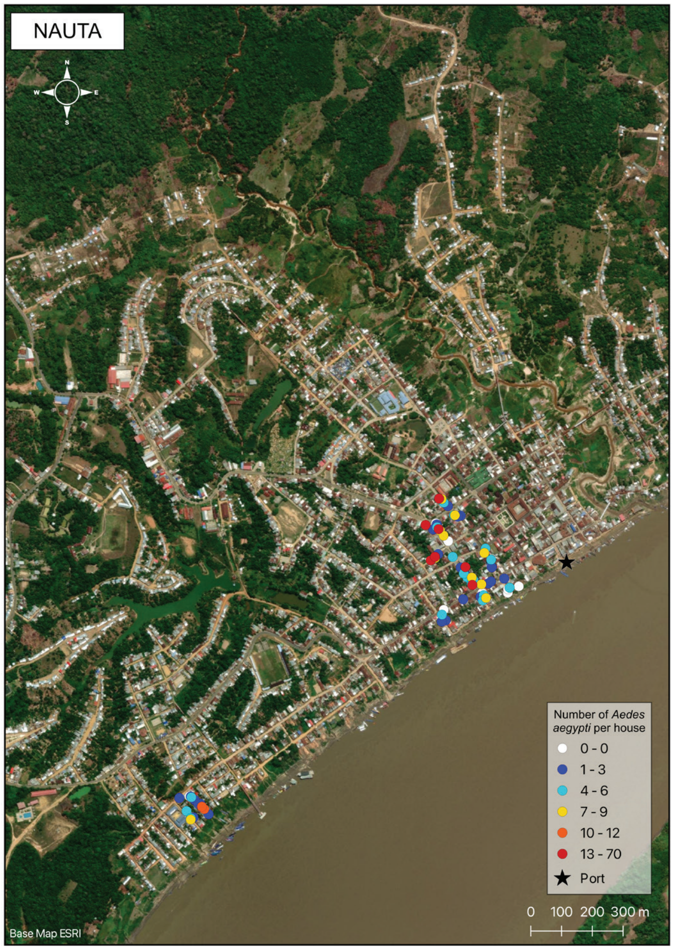

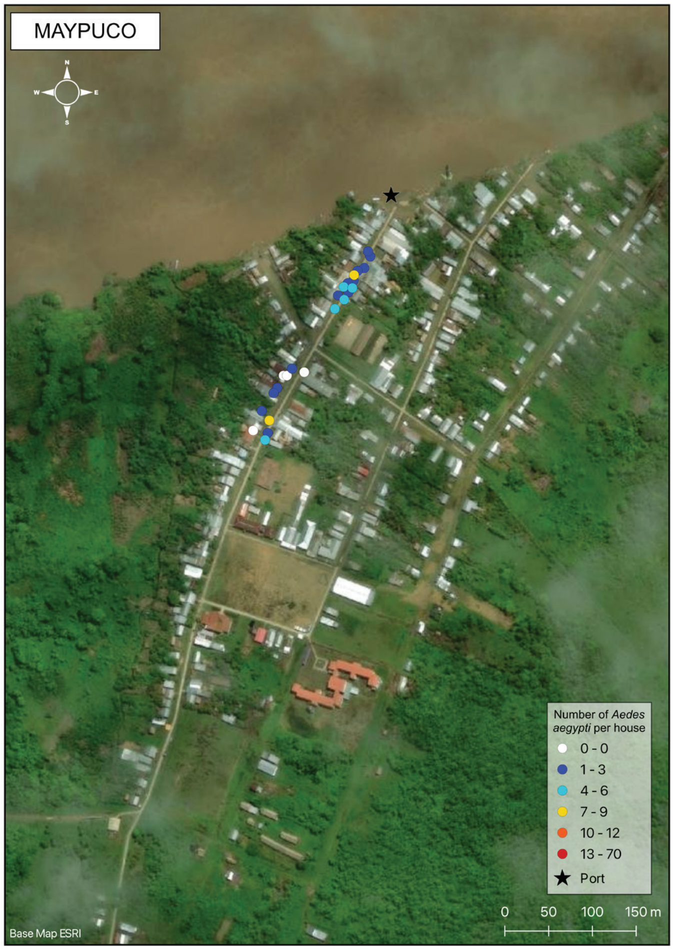

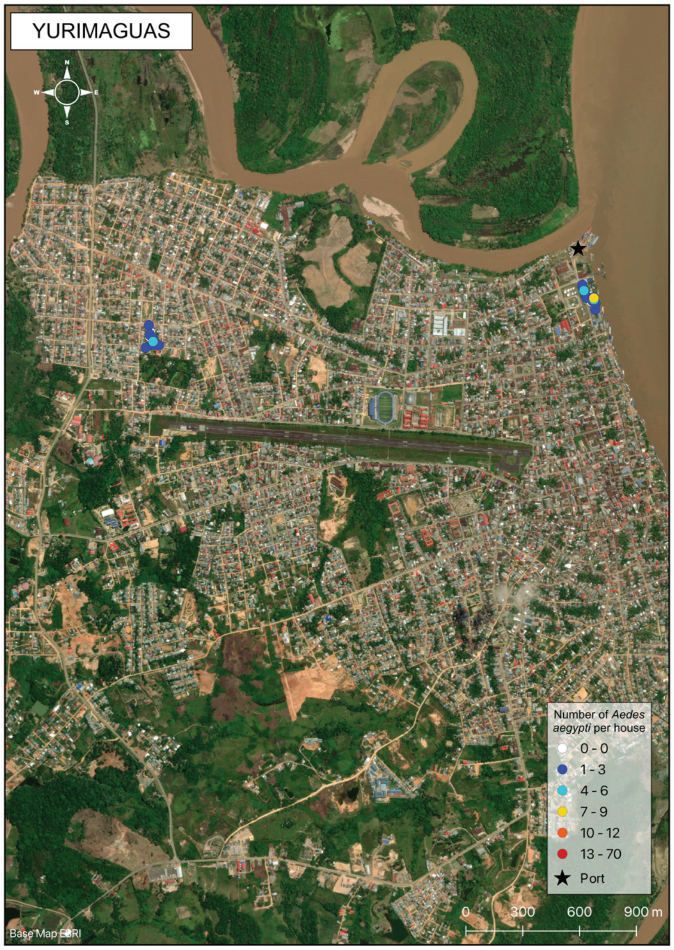

MAPS OF SITES ON THE IQUITOS-NAUTA ROAD

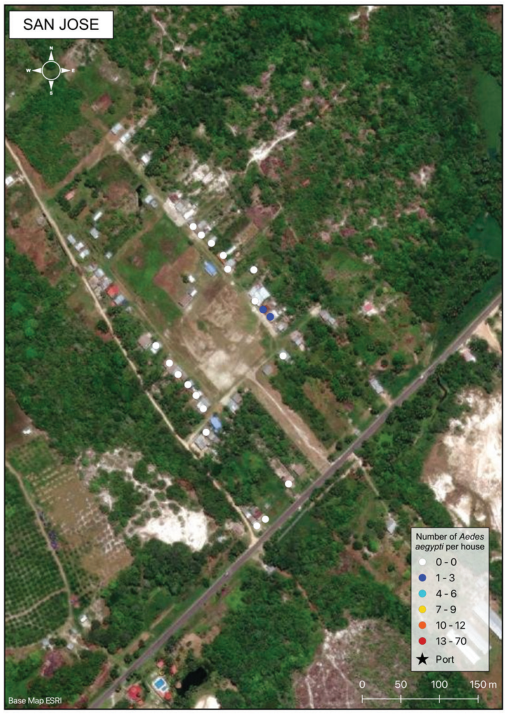

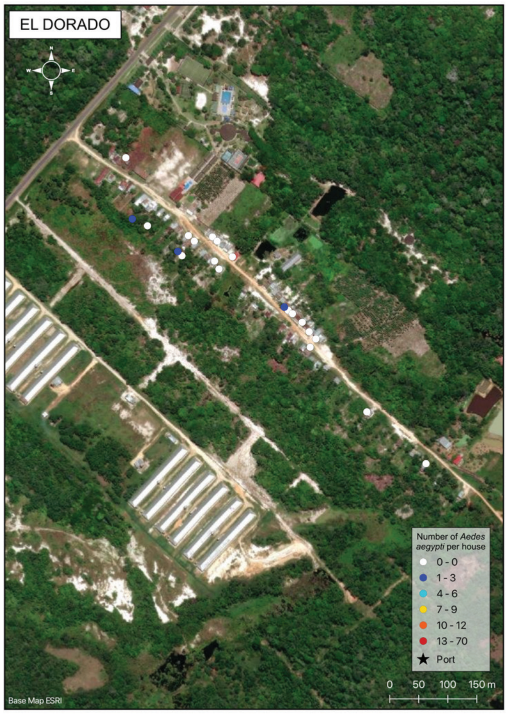

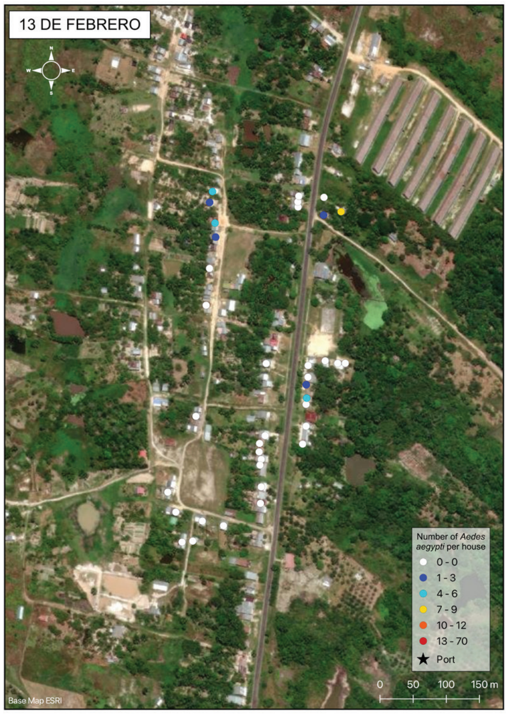

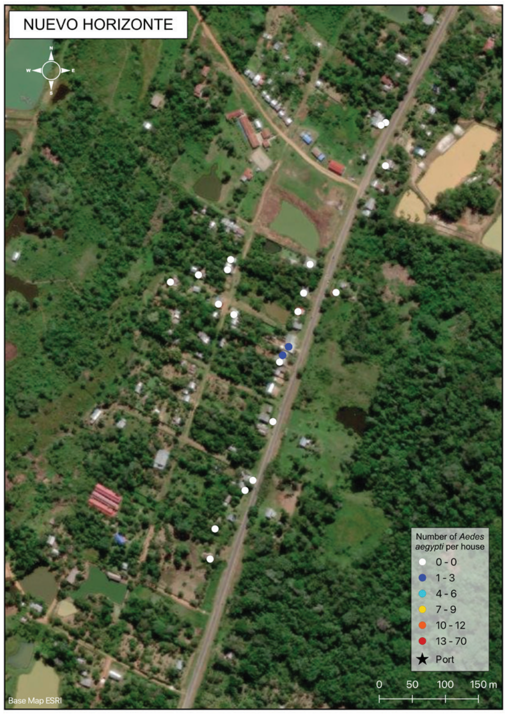

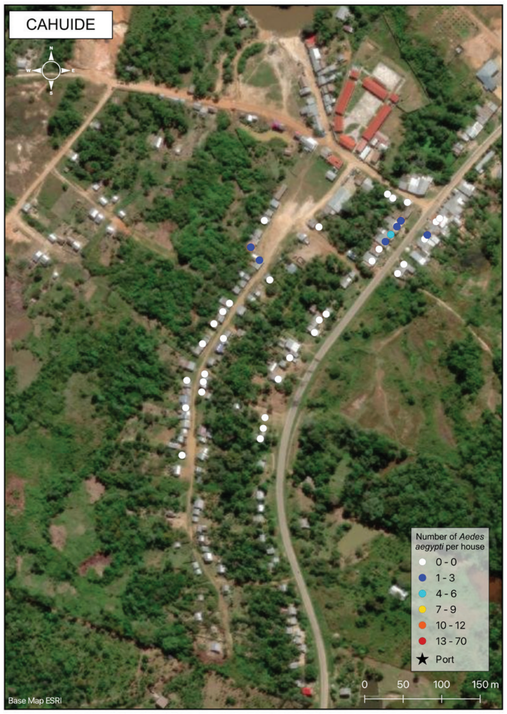

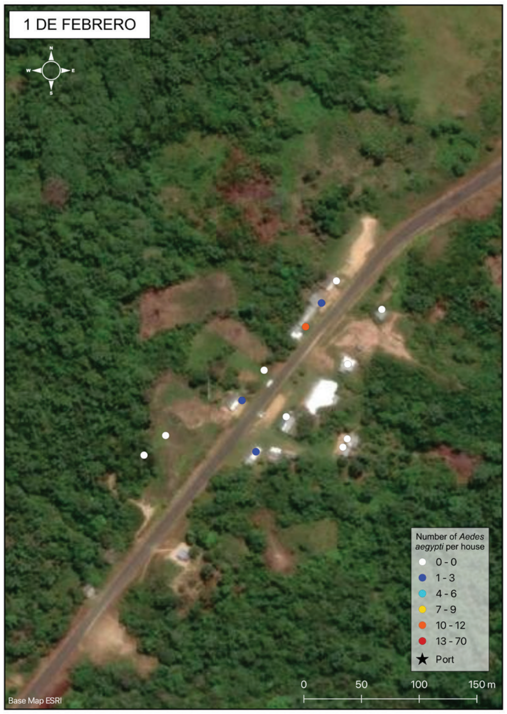

Supplement: S1 Fig — ESRI satellite imagery (2023 ESRI) of the 30 sites, with data points overlaid in qGIS depicting the number of adult Ae. aegypti captured during each household collection event. (PDF) [file pntd.0012506.s007.pdf]
